# Activation of STAT3-mediated ciliated cell survival protects against severe infection by respiratory syncytial virus

**DOI:** 10.1172/JCI183978

**Published:** 2024-11-01

**Authors:** Caiqi Zhao, Yan Bai, Wei Wang, Gaurang M. Amonkar, Hongmei Mou, Judith Olejnik, Adam J. Hume, Elke Mühlberger, Nicholas W. Lukacs, Rachel Fearns, Paul H. Lerou, Xingbin Ai

**Affiliations:** 1 Division of Newborn Medicine, Department of Pediatrics and; 2 The Mucosal Immunology and Biology Research Center, Massachusetts General Hospital for Children, Boston, Massachusetts, USA.; 3 Department of Virology, Immunology & Microbiology, Boston University Chobanian & Avedisian School of Medicine, Boston, Massachusetts, USA.; 4 National Emerging Infectious Diseases Laboratories, Boston University, Boston, Massachusetts, USA.; 5 Mary H. Weiser Food Allergy Center, University of Michigan, Ann Arbor, Michigan, USA.

**Keywords:** Infectious disease, Apoptosis

## Abstract

Respiratory syncytial virus (RSV) selectively targets ciliated cells in human bronchial epithelium and can cause bronchiolitis and pneumonia, mostly in infants. To identify molecular targets of intervention during RSV infection in infants, we investigated how age regulates RSV interaction with the bronchial epithelium barrier. Employing precision-cut lung slices and air-liquid interface cultures generated from infant and adult human donors, we found robust RSV virus spread and extensive apoptotic cell death only in infant bronchial epithelium. In contrast, adult bronchial epithelium showed no barrier damage and limited RSV infection. Single nuclear RNA-Seq revealed age-related insufficiency of an antiapoptotic STAT3 activation response to RSV infection in infant ciliated cells, which was exploited to facilitate virus spread via the extruded apoptotic ciliated cells carrying RSV. Activation of STAT3 and blockade of apoptosis rendered protection against severe RSV infection in infant bronchial epithelium. Lastly, apoptotic inhibitor treatment of a neonatal mouse model of RSV infection mitigated infection and inflammation in the lung. Taken together, our findings identify a STAT3-mediated antiapoptosis pathway as a target to battle severe RSV disease in infants.

## Abstract 2

Susceptibility to severe RSV disease in infants is attributable to an insufficient STAT3 activation response to RSV in bronchial epithelial cells.

## Introduction

Infection of human bronchial epithelium by respiratory syncytial virus (RSV) occurs in people of all ages; however, certain populations, including infants, are at risk of developing severe RSV disease. RSV infection is the leading cause of acute and severe lower airway disease and the primary cause of hospitalization of infants worldwide (1, 2). The pathophysiology of infant RSV disease is characterized by epithelial cell apoptosis and sloughing, inflammation, mucus hyperplasia, and airway obstruction (3, 4). Beyond acute morbidity, RSV infection in infancy is a major risk factor for hyperreactive airway disease and asthma later in life (5–11). To protect the very young from severe RSV disease, decades of intensive effort were spent on vaccine development for infants with disappointing results (12, 13). Very recently, a vaccine for administration during late pregnancy and an RSV antibody (14–17) were clinically approved, offering promising prophylactic strategies against RSV in newborns and infants. However, the challenge of identifying effective treatment strategies for active RSV infection in infants remains.

Most studies of RSV disease in infants focus on the immaturity of the immune system as a contributor to severe infection (3, 18, 19). Apart from immune cells, human bronchial epithelium is the first contact of RSV (20–22) and plays a central role in orchestrating the inflammatory response following respiratory viral infection. The age of the host may regulate the interaction between RSV and bronchial epithelial cells and thus contribute to age-related severity of RSV disease. This possibility is accentuated by the contrasting predilection for severe disease with age following infection by SARS-CoV-2. Like RSV, SARS-CoV-2 also targets ciliated cells (23, 24). However, in contrast to RSV infection, severe COVID-19 disproportionally affects adults, and infants and young children typically have no symptoms after SARS-CoV-2 infection, making them possible spreaders of ongoing and emerging virus variants (25). The drastic difference in clinical outcomes of infants and adults following infection by RSV and SARS-CoV-2 supports the hypothesis that there are virus-specific interactions with human bronchial epithelium that differ between age groups and influence the disease outcome.

Preexisting experimental models have limitations that preclude the elucidation of molecular mechanisms underlying the role of age in RSV infection. A narrow species tropism of RSV creates a major obstacle in modeling infant RSV disease using animal models (26). Generation of RSV strains that are capable of establishing clinically relevant infection in mice helps reduce this technical difficulty (27). The in vitro models, such as immortalized human HEp-2 and A549 cell lines, poorly represent human airway epithelium, which is polarized and contains multiple cell types. To capture the structural and cellular complexity of human bronchial epithelium in vivo, air-liquid interface (ALI) culture of human bronchial basal stem cells (BSCs) is widely used (20, 26, 28). Human bronchial BSCs are traditionally isolated from lung biopsy and airway brushing samples requiring invasive procedures that are often contraindicated in infants. For this reason, prior ALI models of RSV infection utilized bronchial BSCs from older children and adults (20, 28, 29). These previous studies showed that RSV infection has no cytopathic effect on adult epithelial cells in ALI but causes cell sloughing and apoptosis in pediatric ALI cultures (20, 28, 29). To address RSV infection in infants, ALI cultures of nasopharyngeal BSCs, which are more accessible than bronchial BSCs, have been employed (28). However, nasopharyngeal BSCs differentiate into upper respiratory epithelial cells that display lower cytopathic and inflammatory responses to RSV compared with bronchial epithelial cells (28). While previous findings provide evidence for age-related epithelial infection by RSV, specifically, how infant bronchial epithelium responds to RSV, mechanisms determining age-related differences are yet to be discovered.

To generate a clinically relevant human bronchial epithelium model for the investigation of age-related severe RSV disease in infants, we employed an established methodology of BSC derivation from tracheal aspirate (TA) samples (30, 31). TA samples can be collected as part of routine care for intubated patients and are typically considered medical waste. TA contains rare BSCs that can be expanded in culture and differentiated into multiple bronchial epithelial cell types in ALI (30, 31). Importantly, TA BSCs are indistinguishable from bronchial biopsy BSCs in transcriptomic profiles and differentiation potentials and can reproduce bronchial epithelium phenotypes found in vivo, shown by our previous studies of congenital diaphragmatic hernia in newborns (32) and COVID-19 in adults (33). To complement the in vitro bronchial epithelial model in ALI, we have optimized an ex vivo human precision-cut lung slice (hPCLS) model prepared from donor lungs of all ages (34). The hPCLS maintains almost intact airway structure and supports the survival and the function of bronchial epithelial cells for over 1 week in culture (35, 36) and is invaluable for disease modeling and therapeutic testing.

Here, we employed these 2 human bronchial epithelium models of infants and adults to investigate the relationship between age and RSV infection. Our findings reveal that RSV utilizes a virus-specific and STAT3-dependent mechanism of apoptotic cell death to cause severe infection in infant bronchial epithelium. The therapeutic benefit of targeting this mechanism was evaluated in vivo using a neonatal mouse model of RSV infection.

## Results

### RSV causes severe infection of human infant bronchial epithelium in precision-cut lung slice and ALI culture models.

**Figure 1:**
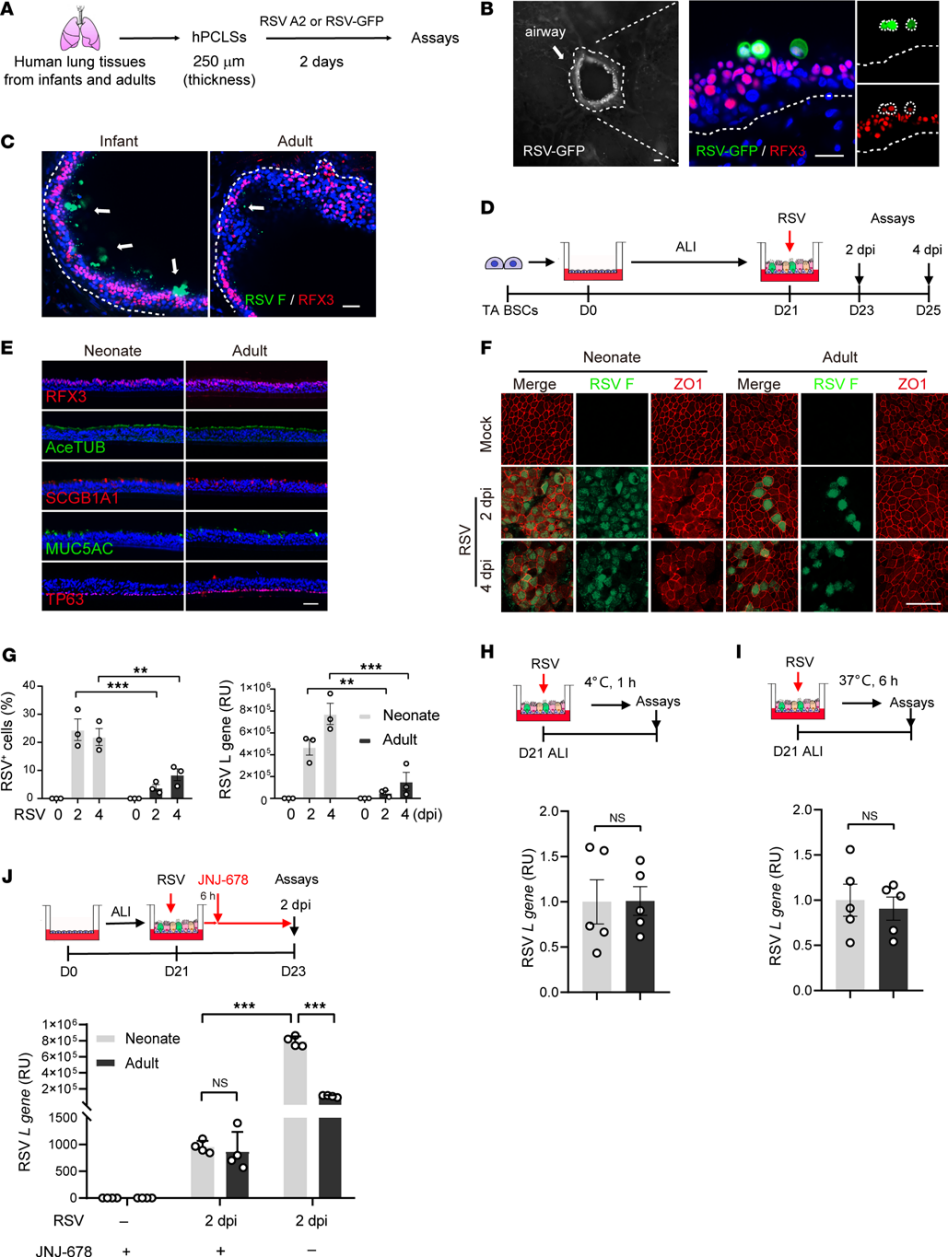
Human bronchial epithelium models generated from hPCLSs and TA BSCs of neonates and adults show age-related RSV infection. (**A**) Schematic of RSV A2 or RSV-GFP infection (1 × 10^6^ pfu) of hPCLSs prepared from donor lungs of infants and adults (*n* = 3 donors) followed by staining and confocal imaging at 2 dpi. (**B**) Representative images of RSV-GFP (green) and RFX3 (red) staining in infant hPCLSs. Left panel shows an RSV infected airway on hPCLS. Arrow indicates an airway infected by RSV. Dotted lines mark basement membrane. Blue color is nucleus staining and the overlay of red, green, and blue shows as pink color. (**C**) Representative images of RSV F (green) and RFX3 (red) staining in infant and adult hPCLSs. Arrows indicate RSV-infected cells. Dotted lines mark basement membrane. Blue color is nucleus staining and the overlay of red, green, and blue shows as pink color. (**D**) Schematic of RSV infection of differentiated bronchial epithelium in day 21 (D21) ALI cultures of neonatal and adult TA BSCs. RSV strain A2 was applied apically (MOI 2, 4 × 10^5^ PFU) for 1 hour. Assays (**E**–**J**) were performed at 2 and 4 dpi. (**E**) Representative cross-section images of antibody staining for major bronchial epithelial cell types prior to infection. Blue color is nucleus staining and the overlay of red, green, and blue shows as pink color. (**F**) Representative top views of double staining for RSV F(green) protein and ZO1 (red). (**G**) Relative abundances of RSV F^+^ cells and relative levels of RSV *L* gene by RT-qPCR. (**H**) Assays of RSV binding at 4°C for 1 hour followed by quantification of relative RSV *L* gene levels by RT-qPCR. (**I**) Assays of primary RSV transcript and genome replication after infection at 37°C for 6 hours by RT-qPCR. (**J**) Assays of JNJ-678 (100 nM) treatment at 6 hpi followed by quantification of relative RSV *L* gene levels by RT-qPCR at 2 dpi. Each dot represents 1 donor. Bar graphs represent mean ± SEM. Statistical significance was calculated by 2-way ANOVA followed by Dunn’s test in **G** and by 2-tailed Student’s *t* test in **H**–**J**. Dotted lines mark basement membrane. **P < 0.01, ***P < 0.001. Scale bars: 50 μm.

To test whether age regulates RSV infection at the human bronchial epithelium barrier, we first employed hPCLSs (250 μm in thickness) generated from infant (0–12 months) and adult (43–69 years) donor lungs as an ex vivo model of infection (Figure 1A). RSV selectively targeted RFX3^+^ ciliated cells in hPCLSs (Figure 1B) as found in vivo. Compared with infant hPCLSs in which more than 10% of bronchial epithelial cells (11.4% ± 1.45%) were infected with RSV at 2 days postinfection (dpi), adult hPCLSs had hardly any RSV^+^ cells (2.13% ± 1.86%, *P* < 0.01) (Figure 1C). Therefore, infant bronchial epithelium is prone to widespread RSV infection.

We then generated human bronchial epithelial models in ALI using infant and adult BSCs to evaluate age-related RSV infection. To circumvent technical difficulties of BSC isolation from infants younger than 6 months of age, we derived BSCs from TA samples of full-term newborns who were intubated primarily for nonpulmonary diseases (Supplemental Table 1; supplemental material available online with this article; https://doi.org/10.1172/JCI183978DS1) (30, 31). Neonatal TA BSCs differentiated into functional epithelial cells in ALI after 21 days (D21) (Figure 1, D and E) (30, 31), a time point when epithelial differentiation of BSCs is considered complete (37, 38). As controls, TA BSCs from adult patients (32–55 years of age) with no known respiratory diseases were similarly derived (Supplemental Table 1) and differentiated in ALI (Figure 1, D and E). Bulk RNA-Seq of neonatal and adult TA BSCs identified differentially expressed genes enriched in the cell cycle pathways (Supplemental Figure 1, A–C), which is consistent with a prior finding that BSC proliferation differs with age (39). We found no age-related difference in the expression of genes involved in epithelial differentiation. Consistently, neonatal and adult TA BSCs exhibited similar differentiation potentials in ALI (Figure 1E and Supplemental Figure 1D). Antibody staining of human donor lungs also showed no difference in the abundance of ciliated cells in the intrapulmonary bronchi of infants and adults (Supplemental Figure 1, E and F). Taken together, age has no apparent effect on the relative abundance of ciliated cells in human bronchial epithelium in vivo or differentiation potentials of BSCs in vitro.

We infected D21 ALI cultures of neonatal and adult TA BSCs with RSV strain A2 (4 × 10^5^ PFU), which was applied apically for 1 hour before saline washes to remove unbound viral particles. Viral load and epithelial responses were assayed at 2 dpi and 4 dpi (Figure 1D). RSV infection of adult ciliated cells elicited no cytotoxicity or barrier damage but induced ciliary dyskinesia (Figure 1F and Supplemental Figure 2, A–D), similar to previous reports (20, 29, 40). In contrast, RSV infection caused cell sloughing and barrier damage in neonatal ALI cultures, manifested by loss of ZO-1, reduced transepithelial electrical resistance (TEER), and crater-like areas in the apical surface (Figure 1F and Supplemental Figure 2, B–D). Compared with adult cultures, neonatal ALI cultures had more RSV-infected cells and higher levels of RSV RNA and expression of inflammatory signals (Figure 1G, Supplemental Figure 2, E and F, and Supplemental Figure 3A). Further, only neonatal ALI cultures showed mucus hyperplasia (Supplemental Figure 3, B–D). Lastly, a clinically isolated human RSV B strain (WV/14617/85) also showed age-related infection in ALI cultures (Supplemental Figure 4). Taken together, RSV infection of both ex vivo and in vitro models of neonatal bronchial epithelium captures clinical hallmarks of severe RSV disease in infants (3, 4).

Notably, BSCs from infant and adult lung biopsy samples generated bronchial epithelium in ALI that showed similar, age-related RSV infection as TA BSCs (Supplemental Figure 2G). This finding, together with the results of our prior studies (31–33), indicate that TA BSCs are bona fide bronchial BSCs. Due to a limited number of lung biopsy BSC lines from infants, we utilized readily available TA BSCs from neonates and adults to generate ALI cultures for mechanistic studies of how age affects epithelial cell interaction with RSV.

### RSV spread in human bronchial epithelium is age related.

To test whether RSV binding to bronchial epithelium differs with age, we incubated RSV with neonatal and adult ALI cultures at 4°C for 1 hour (Figure 1H), a condition that supports RSV binding but not viral entry into host cells. After washes to remove unbound viral particles, viral RNA assays showed that the amount of virus bound to epithelial cells was similar between neonatal and adult ALI cultures (Figure 1H). Therefore, age has no effect on RSV binding to human bronchial epithelial cells.

After viral entry into ciliated cells, RSV undergoes genome expression and replication to generate new virions. At 4–6 hours postinfection (hpi), new viral RNA and protein can be detected intracellularly, but new RSV virions are not yet assembled or released until approximately 12 hpi (22, 41). We thus assayed intracellular viral RNA levels at 6 hpi prior to virus spread and found no difference between neonatal and adult ALI cultures (Figure 1I).

Next, we tested whether age affects the spread of newly released virions. To do so, we treated neonatal and adult ALI cultures with JNJ-678, which blocks RSV fusion (42) and thereby prevents released virions from infecting other cells. JNJ-678 was given at 6 hpi (Figure 1J) to ensure intact RSV entry and primary replication. JNJ-678 effectively prevented virus spread, evidenced by fewer RSV-infected cells and lower RSV RNA levels in both neonatal and adult ALI cultures compared with solvent control (Figure 1J). JNJ-678 treatment completely abrogated the difference in RSV viral RNA levels between neonatal and adult ALI cultures at 2 dpi (Figure 1J). Therefore, age affects the spread of RSV in human bronchial epithelium in ALI.

### Age-related apoptotic cell death causes severe RSV infection in neonatal bronchial epithelium.

**Figure 2:**
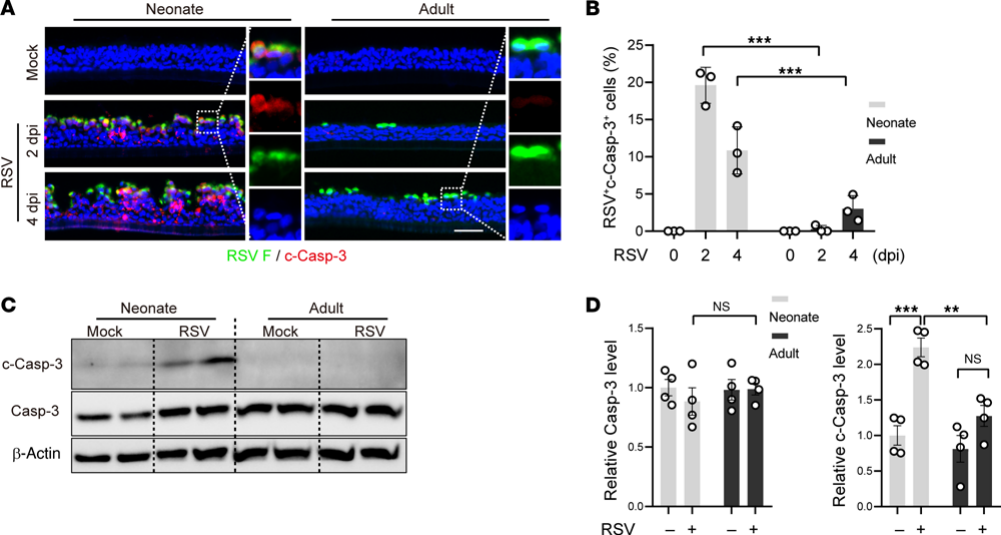
Neonatal bronchial epithelial cells are prone to apoptosis following RSV infection. (**A**) Representative cross-section images of double staining for RSV F (green) and c-Casp-3 (red) in neonatal and adult ALI cultures at 2 and 4 dpi. Blue color is nucleus staining and the overlay of red, green, and blue shows as pink color. (**B**) Relative abundances of RSV F^+^c-Casp-3^+^ cells quantified from double stained images. (**C**) Representative Western blot analyses of Caspase-3 (Casp-3) and c-Casp-3 at 2 dpi. β-actin was loading control. Each lane represents 1 BSC line. (**D**) Densitometry measurements of Casp-3 and c-Casp-3 levels normalized to β-actin. Each dot represents 1 donor. Bar graphs represent mean ± SEM. Statistical significance was calculated by 2-way ANOVA followed by Dunn’s test in **B** and **D**. **P < 0.01, ***P < 0.001. Scale bar: 50 μm.

RSV infection induces bronchial epithelial cell apoptosis in severe disease in infants (3, 4). Given that cell death is a means of virus spread (43), we tested whether age affects apoptosis of RSV-infected ciliated cells and whether apoptosis contributes to virus spread. Staining for cleaved caspase 3 (c-Casp-3) as a marker for apoptosis found negligible apoptotic cell death in neonatal and adult ALI cultures in the absence of infection (Figure 2, A–D). Following RSV infection, c-Casp-3 was barely detectable in adult ALI cultures by antibody staining and Western blot (Figure 2, A–D), consistent with a lack of cytopathy in adult epithelial cells (Figure 1F) (29). In contrast, RSV triggered significant apoptosis in neonatal ALI cultures (Figure 2, A–D). At 2 dpi, approximately 25% of cells in neonatal ALI cultures were RSV F^+^ (fusion glycoprotein) and nearly 80% of these were also c-Casp-3^+^ (Figure 2, A and B). By 4 dpi, the percentage of RSV F^+^c-Casp-3^+^ cells in neonatal ALI cultures was reduced to approximately 10%, likely due to sloughing of RSV-infected cells (Figure 2B). We found an increasing number of c-Casp-3^+^ cells below the apical surface between 2 dpi and 4 dpi that were not infected by RSV (Figure 2A), which may be induced by released inflammatory signals, such as TNF (Supplemental Figure 3A). Similar age-related RSV-induced apoptosis was found in ALI cultures generated from BSCs of infant and adult lung biopsy samples (Supplemental Figure 2G). In addition to apoptosis, a small number of neonatal epithelial cells also expressed necroptotic markers (Supplemental Figure 5), which is consistent with a previous report that RSV infection can induce necroptosis (8). Further, despite similar viral RNA levels in JNJ-678-treated neonatal and adult ALI cultures at 2 dpi (Figure 1J), only neonatal cells were c-Casp-3^+^ (Supplemental Figure 6A). Taken together, neonatal ciliated cells are predisposed to apoptosis following RSV infection, a response that is outgrown in adults.

**Figure 3:**
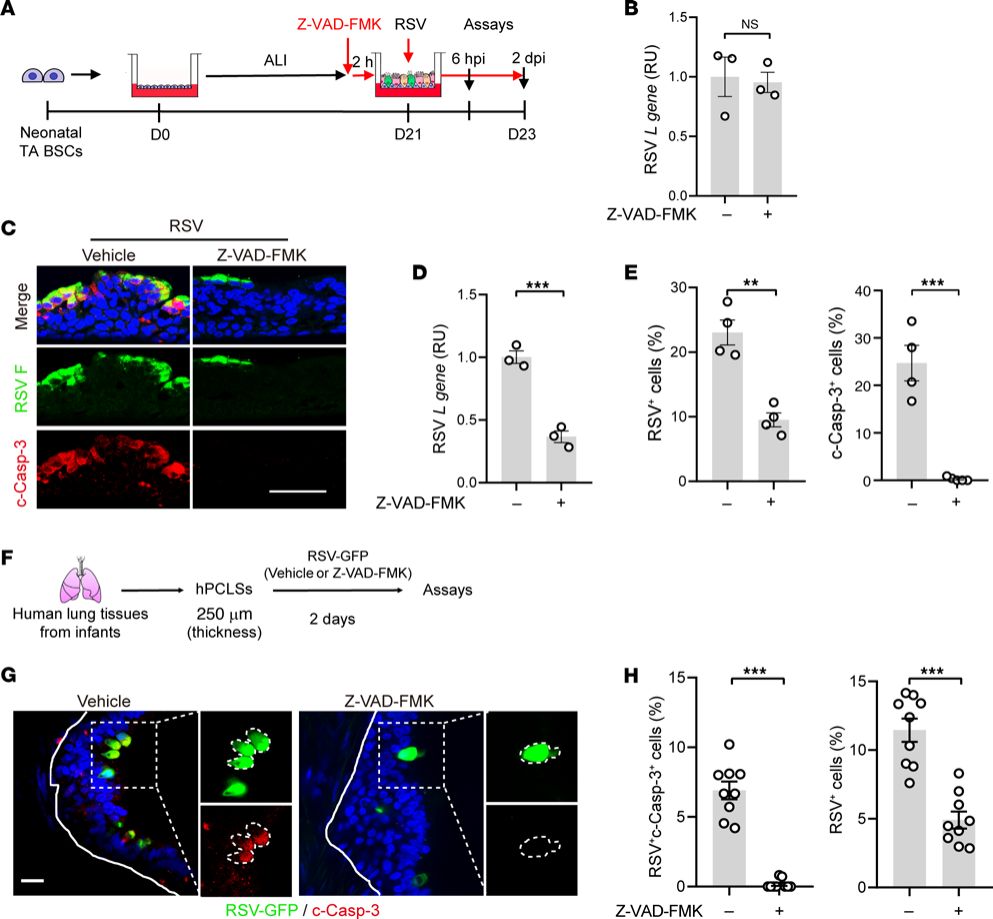
Age-related apoptotic cell death causes severe RSV infection in neonatal bronchial epithelium. (**A**) Schematic of Z-VAD-FMK treatment of neonatal ALI cultures. Z-VAD-FMK (40 μM) was applied in the bottom chamber 2 hours prior to RSV infection until the assays performed at 6 hpi and 2 dpi in **B**–**E**. (**B**) Relative levels of RSV *L* gene at 6 hpi by RT-qPCR. (**C**) Representative double staining for RSV F (green) and c-Casp-3 (red) at 2 dpi. Blue color is nucleus staining and the overlay of red, green, and blue shows as pink color. (**D**) Relative levels of RSV *L* gene at 2 dpi by RT-qPCR. (**E**) Relative abundances of RSV F^+^ and c-Casp-3^+^ cells at 2 dpi quantified from double stained images. (**F**) Schematic of RSV-GFP infection (1 × 10^6^ pfu) of infant hPCLSs pretreated with vehicle or Z-VAD-FMK (40 μM) 2 hours prior to infection. (**G**) Representative images of RSV-GFP (green) and c-Casp-3 (red) staining in control and Z-VAD-FMK–treated infant hPCLSs. The contour of RSV-GFP^+^ cells was outlined in panels showing enlarged areas. Blue color is nucleus staining and the overlay of red, green, and blue shows as pink color. Solid lines mark basement membrane and dotted lines mark the enlarged areas. (**H**) Relative abundance of RSV-GFP^+^ cells and RSV^+^c-Casp-3^+^ cells in bronchial epithelium of infant hPCLSs. Each dot represents quantification of 1 airway. A total of 9 airways from 2 donors, 4–5 airways per donor, were quantified. Each dot (except for panel **H**) represents 1 donor. Bar graphs represent mean ± SEM. Statistical significance was calculated by 2-tailed Student’s *t* test in **B**, **D**, **E**, and **H**. Solid lines mark basement membrane. **P < 0.01, ***P < 0.001. Scale bar: 50 μm.

To assess whether apoptosis facilitates RSV spread, we treated RSV-infected neonatal ALI cultures with a caspase inhibitor, Z-VAD-FMK, starting 2 hours before infection (Figure 3A). At 6 hpi, the viral RNA level was comparable between solvent- and Z-VAD-FMK–treated neonatal ALI cultures (Figure 3B). At 2 dpi, Z-VAD-FMK treatment prevented apoptosis in neonatal epithelial cells and reduced the number of RSV-infected cells and viral RNA by more than 50% compared with the solvent control (Figure 3, C–E). Z-VAD-FMK treatment also ameliorated mucus hyperplasia and decreased the level of inflammatory gene expression in RSV-infected neonatal ALI cultures (Supplemental Figure 6, B and C). A second caspase 3 inhibitor, Z-DEVD-FMK, had similar activities (Supplemental Figure 6, D and E). Further, RSV-infected ciliated cells in infant hPCLSs underwent apoptosis shown by antibody staining for c-Casp-3 (Figure 3, F and G), and Z-VAD-FMK treatment reduced apoptosis and the abundance of RSV^+^ ciliated cells (Figure 3, G and H). In contrast, adult hPCLSs had hardly any RSV^+^ cells, none of which were c-Casp-3^+^ (Supplemental Figure 7A). These findings indicate that apoptosis of RSV-infected ciliated cells promotes virus spread.

### RSV-infected ciliated cells can spread RSV after extrusion from bronchial epithelium.

**Figure 4:**
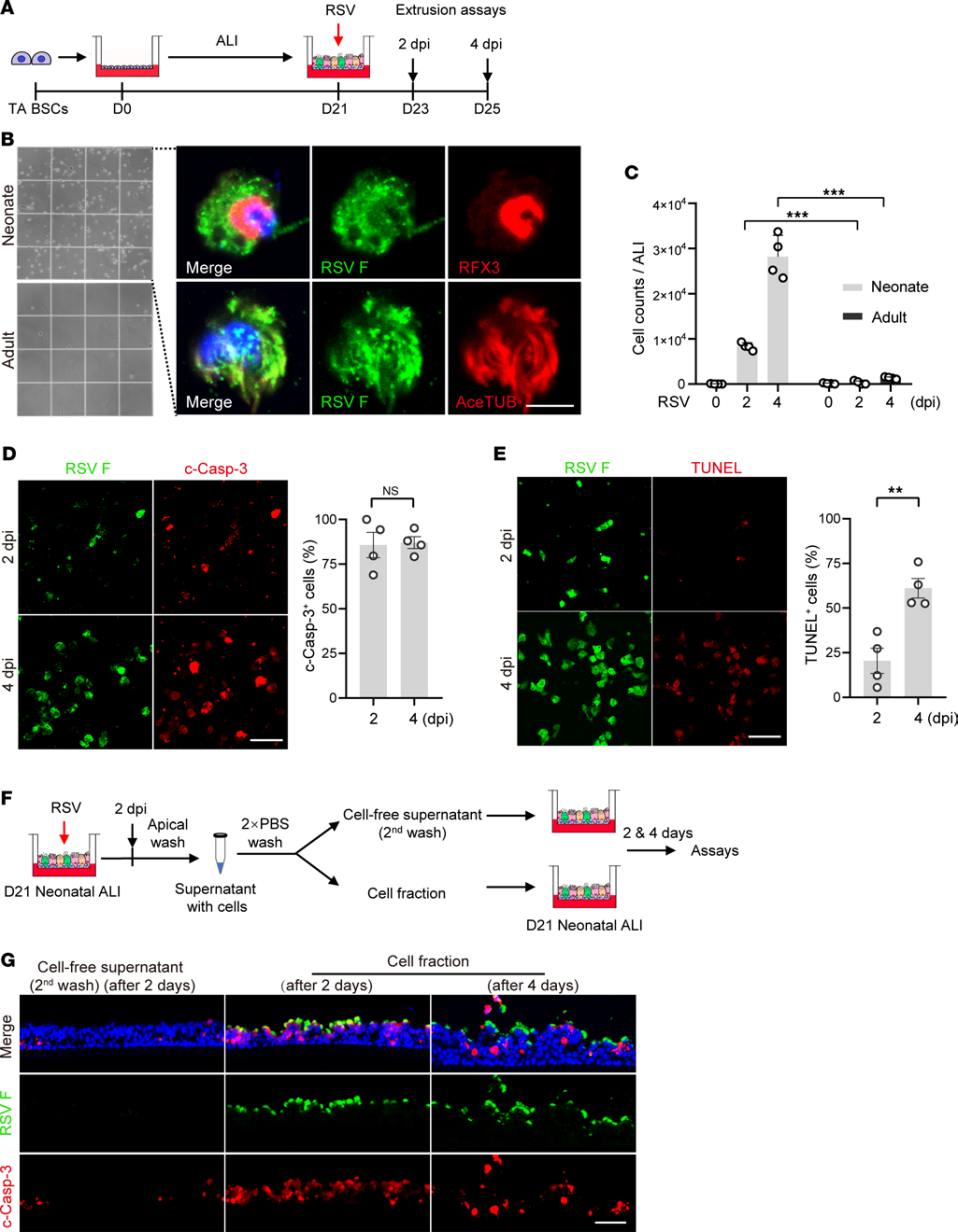
Apoptotic cell extrusion in neonatal epithelium model following RSV infection can mediate virus spread. (**A**) Schematic of extrusion assays. Extruded cells were collected from apical washes of neonatal and adult ALI cultures at 2 and 4 dpi and analyzed in **B**–**E**. (**B**) Representative images of extruded cells on a cytometer (left panels) and double staining for RSV F protein (green) and ciliated cell markers (RFX3 and AceTUB [red])Blue color is nucleus staining and the overlay of red, green, and blue shows as pink color.) (right panels). (**C**) Quantification of the number of extruded cells. (**D**) Representative double staining for RSV F (green) protein and c-Casp-3 (red) (left panels) and quantification of the relative abundance of c-Casp-3^+^ cells (right panel). (**E**) Representative double staining for RSV F (green) protein and TUNEL(red) (left panels) and quantification of the relative abundance of TUNEL^+^ cells (right panel). (**F**) Schematic of infection assay by extruded cells. (**G**) Representative double staining for RSV F (green) and c-Casp-3 (red) after treatment with cell fraction and cell-free supernatant. Blue color is nucleus staining and the overlay of red, green, and blue shows as pink color. Each dot represents 1 donor. Bar graphs show mean ± SEM. ***P < 0.001 calculated by 2-way ANOVA followed by Dunn’s test in **C**. **P < 0.01 by 2-tailed Student’s *t* test in **D** and **E**. Scale bars: 5 μm (**B**) and 50 μm (**D**, **E**, and **G**).

Mucosal epithelium extrudes apoptotic cells to maintain the integrity of barrier function (44, 45). To test whether extrusion of apoptotic RSV-infected ciliated cells can facilitate virus spread, we collected extruded cells in apical washes of neonatal ALI cultures (Figure 4A). The extruded cells were RSV-infected ciliated cells (Figure 4B) and the cell number increased by more than 3-fold between 2 dpi and 4 dpi (Figure 4C). Almost all extruded cells were c-Casp3^+^, indicating ongoing apoptosis (Figure 4D). In contrast, few epithelial cells were extruded in RSV-infected adult ALI cultures (Figure 4, B and C), consistent with a lack of apoptosis in these cultures (Figure 2) (29). TUNEL staining of extruded neonatal cells for fragmented DNA, a marker of the final stage of apoptotic cell death, showed an increase in the abundance of terminally dying cells from less than 20% at 2 dpi to approximately 60% at 4 dpi (Figure 4E). These findings are consistent with extrusion of epithelial cells during the early phase of apoptosis (46, 47) and the ongoing process of apoptotic cell death in extruded ciliated cells provides a time window for these cells to spread RSV.

To test whether extruded ciliated cells can spread RSV, we collected the cell fraction from apical washes of neonatal ALI cultures at 2 dpi by centrifugation (Figure 4F). After 2 rounds of saline washes to remove extracellular virions, cell pellets were resuspended in saline and the cell-free supernatant from the second wash was collected (Figure 4F). The cell fraction applied apically to uninfected neonatal ALI cultures overnight was able to induce widespread RSV infection after 2 and 4 days (Figure 4, F and G). This was not due to carryover of already-secreted RSV virions because the cell-free supernatant failed to establish infection (Figure 4G). Therefore, extruded, RSV-infected ciliated cells can spread RSV during the process of apoptotic cell death.

Consistent with our findings in ALI cultures, RSV-infected ciliated cells in infant hPCLSs exhibited a partial or complete loss of cilia (Supplemental Figure 7B). Those with severe ciliary damage underwent morphology changes from a cuboidal to waterdrop shape with the nucleus translocating from the basal side to the apical side of the cell, indicative of cell extrusion (Supplemental Figure 7B). In contrast, RSV-infected ciliated cells in Z-VAD-FMK–treated infant hPCLSs retained cilia and maintained the cuboidal cell morphology (Supplemental Figure 7B). These findings support that RSV exploits extrusion of apoptotic ciliated cells to exacerbate viral infection in infant bronchial epithelium.

### RSV induces age-related changes in gene expression in ciliated cells.

**Figure 5:**
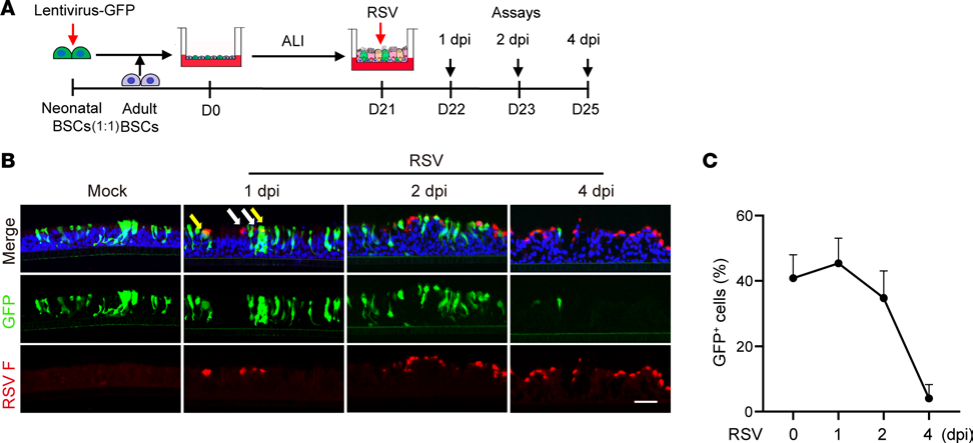
Age regulates bronchial epithelial cell survival following RSV infection in a cell-autonomous manner. (**A**) Schematic of RSV infection of a hybrid ALI culture. Hybrid ALI cultures established with GFP-lentivirus transduced neonatal BSCs and empty-lentivirus transduced adult BSCs were analyzed at 1, 2, and 4 dpi in **B** and **C**. *n* = 2 donors. (**B**) Representative staining for RSV F (red) protein in hybrid ALI cultures. White arrows mark RSV F^+^GFP^–^ (adult) cells and yellow arrows mark RSV F^+^GFP^+^ (green) (neonatal) cells. Blue color is nucleus staining. Scale bar: 50 μm. (**C**) The relative abundance of GFP^+^ cells in hybrid ALI cultures.

We tested whether age autonomously regulates RSV infection of human bronchial epithelium by generating a hybrid bronchial epithelium model in ALI from mixed neonatal and adult BSCs at a 1:1 ratio (Figure 5A). Neonatal BSCs were prelabeled by a GFP lentivirus to track their progeny and adult BSCs were transduced by an empty lentivirus (Figure 5, A and B). GFP^+^ (neonatal) and GFP^–^ (adult) ciliated cells were found at an equal frequency in the apical surface of hybrid ALI cultures at D21 and had similar rates of RSV infection at 1 dpi (Figure 5, B and C). The number of RSV^+^GFP^+^ cells (neonatal) was reduced at 2 dpi, and by 4 dpi, only a few GFP^+^ cells remained (Figure 5, B and C), which is similar to the time course of neonatal cell apoptosis in neonatal (only) ALI cultures following RSV infection (Figure 2B). Therefore, the presence of adult epithelial cells imparted no protection from apoptosis to neonatal epithelial cells, which supports a cell-autonomous role of age in regulating RSV-induced apoptosis. At 4 dpi, compared with adult (only) ALI cultures in which approximately 8% of epithelial cells were RSV^+^ (Figure 1G), hybrid cultures had up to 15% of RSV^+^GFP^–^ cells (adult) despite a significant loss of initially infected neonatal cells (Figure 5, B and C). These findings provide further evidence for apoptosis-mediated virus spread from RSV-infected neonatal epithelial cells to neighboring adult cells.

**Figure 6:**
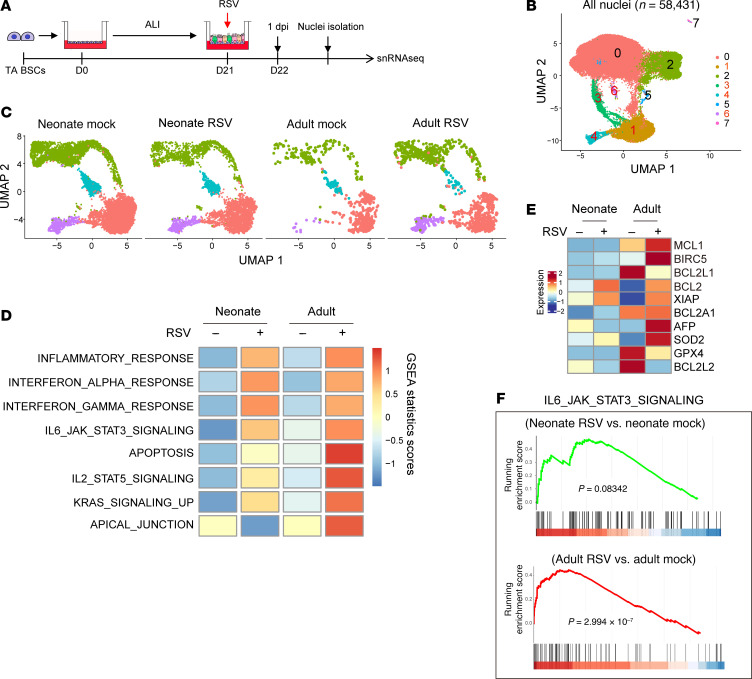
RSV induces age-related changes in expression of genes involved in cell survival and STAT3 signaling in ciliated cells. (**A**) Schematic of snRNA-Seq of neonatal and adult ALI cultures with mock and RSV infection at 1 dpi. (**B**) Combined UMAP plot showing all nuclei (*n* = 58,431) in 8 clusters. (**C**) UMAP plots showing mock- and RSV-infected, neonatal and adult ciliated cells (*n* = 14,464). (**D**) Heatmap showing enrichment of different pathways in ciliated cells of the 4 experimental groups by single-cell GSEA. (**E**) Heatmap showing relative expression of antiapoptotic genes in neonatal and adult ciliated cells. (**F**) Enrichment plots for RSV-induced changes in expression of IL6_JAK_STAT3_SIGNALING pathway genes between neonatal and adult ciliated cells.

To identify the mediator of age that acts autonomously to regulate ciliated cell apoptosis following RSV infection, we conducted single-nuclei RNA sequencing (snRNA-Seq) of neonatal and adult ALI cultures at 1 dpi (Figure 6A), an early time point when RSV infection was comparable between the 2 ages (Supplemental Figure 2E) and no significant cell death occurred in neonatal ALI cultures. snRNA-Seq circumvented technical difficulties of isolating live ciliated cells from differentiated airway epithelium, which were likely encountered by prior single-cell RNA-Seq resulting in a relatively low number of ciliated cells (37, 48). A total of 58,431 nuclei were sequenced from the 4 experimental groups (Neonate Mock, Neonate RSV, Adult Mock, and Adult RSV) that generated 8 cell clusters in UMAP (Figure 6B). Based on established airway epithelial cell markers, we designated cluster 0 as secretory and differentiating cells (33,809 nuclei), clusters 1, 3, 4, and 6 as ciliated cells (14,464 nuclei), cluster 2 as BSCs (9,673 nuclei), cluster 5 as tuft cells (373 nuclei), and cluster 7 as ionocytes (112 nuclei) (Supplemental Figure 8, A and B). The 4 clusters of ciliated cells are consistent with the presence of multiple subtypes of ciliated cells previously identified in lungs and ALI cultures (Supplemental Figure 8B) (37, 49, 50). Because the RSV genome does not enter the nucleus and no distinct cell cluster emerged following infection (Figure 6C), we were unable to identify specific ciliated cells that were infected with RSV by snRNA-Seq. Gene set enrichment analysis (GSEA) of all ciliated cells between mock and RSV infection identified similar inflammatory and interferon responses at 1 dpi independent of age (Figure 6D), indicating that neonatal ciliated cells have already matured in RSV-sensing and early antiviral responses. However, genes involved in the IL6-JAK-STAT3, apoptosis, IL2-STAT5, KRAS, and apical junction pathways showed age-related changes at baseline and in response to RSV infection (Figure 6D and Supplemental Figure 8, C and D). A number of antiapoptotic genes, including the BCL2 family, had lower levels of expression at baseline or showed smaller fold changes following infection in neonatal ciliated cells compared with adult ciliated cells (Figure 6E), which is consistent with prevalent apoptosis observed in neonatal ALI cultures at 2 dpi and 4 dpi (Figure 2). In addition, only adult ciliated cells had significant enrichment in STAT3 signaling genes in response to RSV infection (Figure 6F). STAT3 is a transcriptional factor involved in innate immunity (51) and plays a critical role in cell survival by elevating the transcription of antiapoptotic target genes within the *BCL2* family (52–54). These findings suggest that age-related STAT3 activation in response to RSV infection may drive a transcriptional program against apoptosis and possibly other damaging effects of RSV.

### Age affects STAT3 activation in ciliated cells following RSV infection.

**Figure 7:**
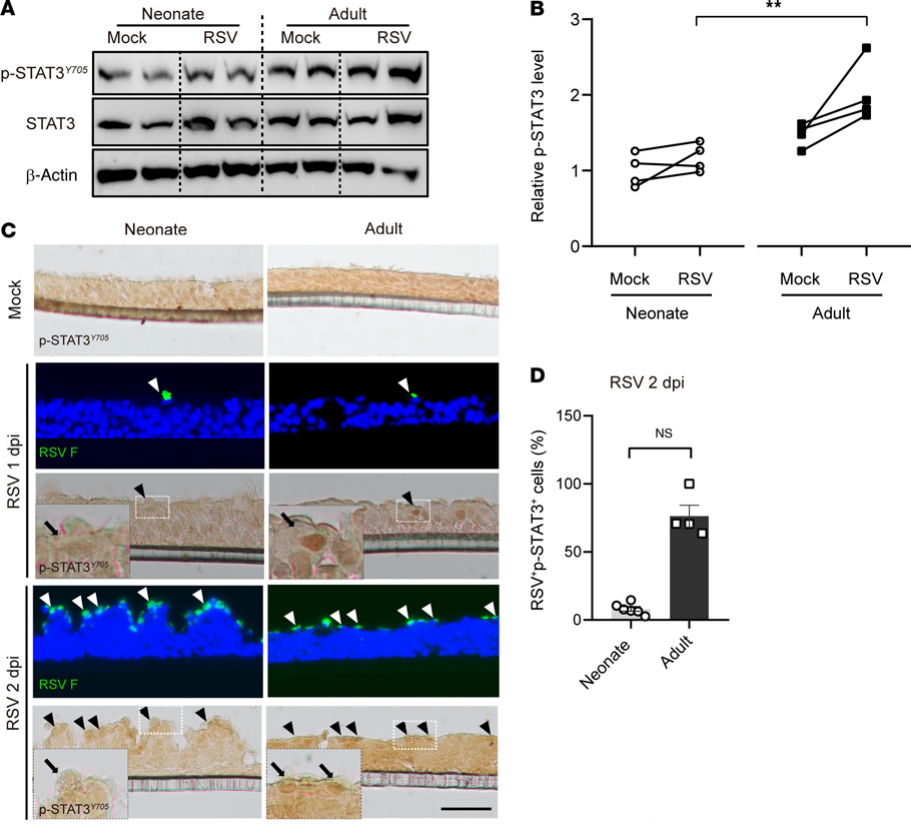
Age regulates STAT3 activation following RSV infection in ciliated cells. (**A**) Representative Western blot analyses of p-STAT3^Y705^ and STAT3 levels at 2 dpi in neonatal and adult ALI cultures. β-Actin was loading control. Each lane represents 1 BSC line. (**B**) Densitometry measurements of relative levels of p-STAT3^Y705^ for individual BSC line before RSV infection (mock) and at 2 dpi. *n* = 4 donors for each age. **P < 0.01 by 2-way ANOVA followed by Dunn’s test. (**C**) Representative double staining for RSV F (fluorescence) (green) and p-STAT3^Y705^ (chromogenic). Blue color is nucleus staining. Arrowheads mar RSV F^+^ ciliated cells. Inserts show enlarged images of p-STAT3^Y705^ staining. Scale bar: 50 μm. (**D**) Quantification of the percentage of double RSV^+^p-STAT3^+^ cells among RSV-infected epithelial cells in neonatal and adult ALI cultures. Each dot represents 1 donor. **P < 0.01 by Student’s *t* test.

Guided by the results of snRNA-Seq, we measured the level of total STAT3 and activated STAT3 (p-STAT3^Y705^) in neonatal and adult ALI cultures prior to and following RSV infection. Western blot showed similar levels of total STAT3 between the 2 ages regardless of RSV infection (Figure 7A). The relative level of p-STAT3^Y705^ before infection was approximately 30% higher in adult ALI cultures than neonatal ALI cultures (Figure 7, A and B). Antibody staining showed nuclear p-STAT3^Y705^ uniformly distributed in uninfected, neonatal, and adult ALI cultures at low levels, as the signal was detected only after extended chromogenic exposure (Figure 7C).

Following RSV infection at 1 dpi and 2 dpi, nuclear p-STAT3 became readily detectable only in adult ciliated cells that were infected with RSV by antibody staining (Figure 7, C and D). Western blot also showed that only adult ALI cultures elevated p-STAT3^Y705^ levels above an already higher baseline (Figure 7, A and B). Given age-related differences in the number of RSV-infected cells at 2 dpi (Figure 1G), the p-STAT3^Y705^ level per RSV-infected ciliated cell was estimated to be minimally 5-fold higher in adult ALI cultures than neonatal ALI cultures. Further, STAT3^Y705^ levels correlated with age-related differences in BCL2 family gene expression in ALI cultures at baseline and following RSV infection (Supplemental Figure 9A).

Like RSV, SARS-CoV-2 also targeted ciliated cells in ALI cultures (Supplemental Figure 10, A, B, and E) (23, 24). However, there was no difference in the number of SARS-CoV-2–infected cells between neonatal and adult ALI cultures (Supplemental Figure 10, B–D). In addition, we found no evidence for apoptotic cell death or STAT3 activation following SARS-CoV-2 infection in either age group (Supplemental Figure 10, E and F). Therefore, RSV utilizes virus-specific regulation of STAT3 signaling and apoptotic cell death to induce severe infection in neonatal bronchial epithelium.

### STAT3 inhibition in adult bronchial epithelium model promotes apoptosis and worsens RSV infection.

**Figure 8:**
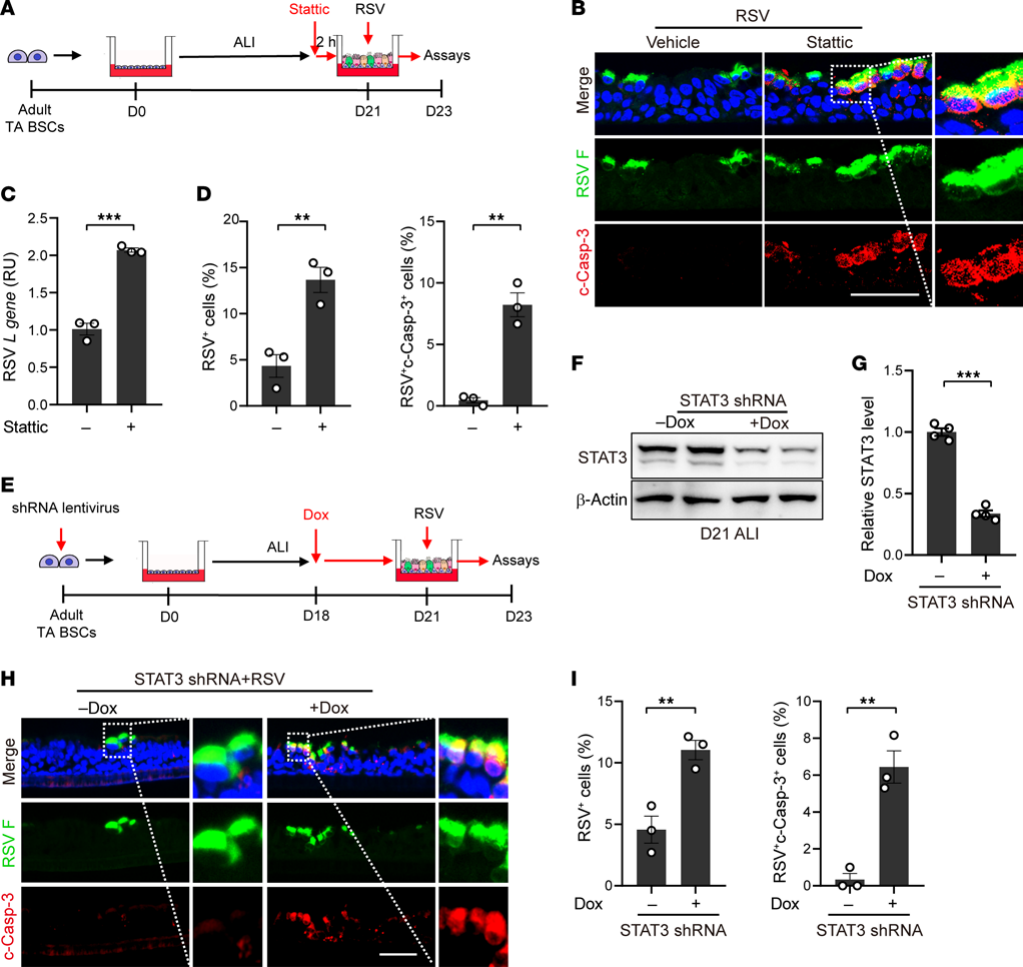
Blockade of active STAT3 in RSV-infected adult bronchial epithelium model worsens infection and promotes apoptosis. (**A**) Schematic of stattic (20 μM) treatment. Stattic was applied in the bottom chamber of adult ALI cultures 2 hours prior to RSV infection until 2 dpi. Results were shown in **B**–**D**. (**B**) Representative double staining for RSV F (green) protein and c-Casp-3 (red). Blue color is nucleus staining and the overlay of red, green, and blue shows as pink color. (**C**) The relative level of RSV *L* gene by RT-qPCR. (**D**) The relative abundance of RSV F^+^ and RSV F^+^c-Casp-3^+^ cells. (**E**) Schematic of STAT3 knockdown assay using an inducible lenti-shRNA system. Dox (500 ng/mL) was added to the bottom chamber from day 18. Assays were performed 2 dpi and results were shown in **F**–**H**. (**F**) Representative Western blot analyses to assess STAT3 knockdown efficiency. β-actin was loading control. Each lane represents 1 BSC line. (**G**) Densitometry measurements of STAT3 levels normalized to β-actin. (**H**) Representative double staining for RSV F (green) protein and c-Casp-3 (red). Blue color is nucleus staining and the overlay of red, green, and blue shows as pink color. Dotted lines show area indicated in enlarged images. (**I**) Quantification of the relative abundance of RSV F^+^ cells and RSV F^+^c-Casp-3^+^ cells. Each dot represents 1 donor. **P < 0.01 and ***P < 0.001 calculated by 2-tailed Student’s *t* test. Scale bars: 50 μm.

To functionally link age-related STAT3 activation to apoptosis and RSV spread, we inhibited STAT3 activity in adult ALI cultures using 3 different approaches. Firstly, we treated adult ALI cultures with a specific STAT3 inhibitor, stattic, 2 hours before RSV infection until 2 dpi (Figure 8A). The dose of stattic (20 μM) was determined based on its efficacy in blocking the increase in p-STAT3^Y705^ levels in response to IL6 (Supplemental Figure 11A). Stattic treatment had no effect on the expression of an RSV receptor gene known to be expressed by ciliated cells, *IGF1R* (55) (Supplemental Figure 11B) or barrier function of bronchial epithelium in adult ALI cultures (56). At 2 dpi, stattic treatment doubled the number of RSV-infected cells and the viral RNA level compared with the solvent control and induced apoptosis in approximately 8% of epithelial cells that were otherwise fully protected (Figure 8, B–D). Secondly, we employed a doxycycline-inducible (Dox-inducible) shRNA system to knockdown STAT3. Dox was administered from D18 in ALI (Figure 8E), when epithelial cell lineages are already committed (37, 38), to minimize the effect of STAT3 inhibition on ciliated cell differentiation (56, 57). At D21 in ALI, STAT3 levels were reduced by more than 60% by Dox (Figure 8, F and G). STAT3 knockdown in adult ALI cultures resulted in almost 3 times more RSV-infected cells, among which approximately 60% were apoptotic (Figure 8, H and I). Thirdly, we isolated bronchial BSCs from lung biopsy samples from an adult patient with Job syndrome harboring a hypomorphic STAT3-S560del mutation (56) (Supplemental Figure 12A). STAT3-S560del BSCs had a reduced capacity to differentiate into ciliated cells compared with healthy biopsy BSCs (Supplemental Figure 12, B and C) (56). However, despite fewer target cells for RSV, STAT3-S560del adult ALI cultures had more RSV F^+^c-Caps-3^+^ cells than adult controls (Supplemental Figure 12, D and E). All 3 approaches to inhibit STAT3 reduced *BCL2* gene expression in adult ALI cultures (Supplemental Figure 9, B and C), consistent with *BCL2* being a direct target gene of activated STAT3 (52, 53). Taken together, STAT3 activation in adult epithelial cells following RSV infection is required to prevent apoptosis and reduce severity of infection.

### STAT3 activation in neonatal bronchial epithelial cells reduces severity of RSV infection.

**Figure 9:**
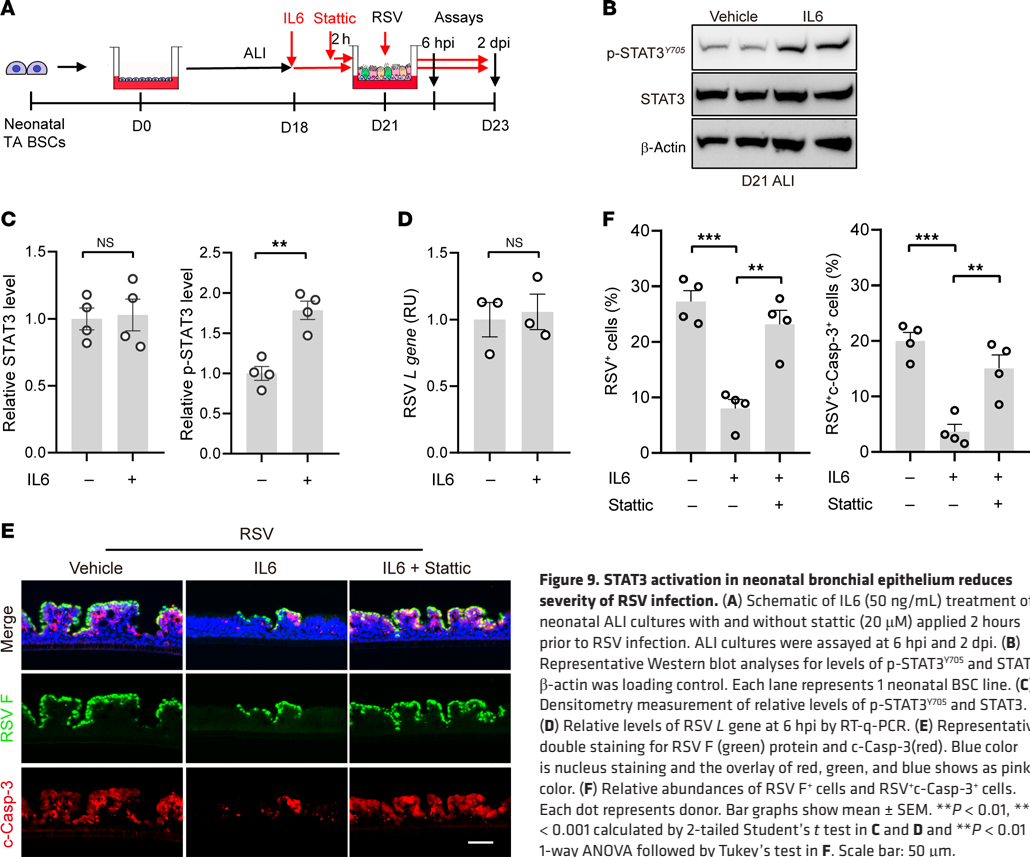
STAT3 activation in neonatal bronchial epithelium reduces severity of RSV infection. (**A**) Schematic of IL6 (50 ng/mL) treatment of neonatal ALI cultures with and without stattic (20 μM) applied 2 hours prior to RSV infection. ALI cultures were assayed at 6 hpi and 2 dpi. (**B**) Representative Western blot analyses for levels of p-STAT3^Y705^ and STAT3. β-actin was loading control. Each lane represents 1 neonatal BSC line. (**C**) Densitometry measurement of relative levels of p-STAT3^Y705^ and STAT3. (**D**) Relative levels of RSV *L* gene at 6 hpi by RT-q-PCR. (**E**) Representative double staining for RSV F (green) protein and c-Casp-3(red). Blue color is nucleus staining and the overlay of red, green, and blue shows as pink color. (**F**) Relative abundances of RSV F^+^ cells and RSV^+^c-Casp-3^+^ cells. Each dot represents donor. Bar graphs show mean ± SEM. **P < 0.01, ***P < 0.001 calculated by 2-tailed Student’s *t* test in **C** and **D** and **P < 0.01 by 1-way ANOVA followed by Tukey’s test in **F**. Scale bar: 50 μm.

To test whether augmenting STAT3 activation in neonatal epithelial cells can mitigate severe RSV infection, neonatal ALI cultures were treated with IL6 at D18 in ALI until 2 dpi (Figure 9A). The timing and the duration of IL6 treatment were determined based on the efficacy of increasing p-STAT3 levels and *BCL2* gene expression (Figure 9, B and C and Supplemental Figure 9D) while preserving mucociliary differentiation in neonatal ALI cultures (Supplemental Figure 13). We showed that IL6 treatment had no effect on early events of RSV infection assayed at 6 hpi (Figure 9D). At 2 dpi, IL6 treatment significantly reduced the number of RSV-infected and apoptotic ciliated cells to a similar level as RSV-infected adult ALI cultures (Figure 9, E and F compared with Figure 1G and 2B). The beneficial effect of IL6 was blunted by overlapping stattic blockade during RSV infection (Figure 9, A, E, and F), indicating active STAT3 as the mediator of IL6 treatment. Taken together, insufficient STAT3 activation is a major cause of severe RSV infection in neonatal bronchial epithelium.

### Blockade of apoptosis ameliorates RSV infection and lung inflammation in neonatal mice.

**Figure 10:**
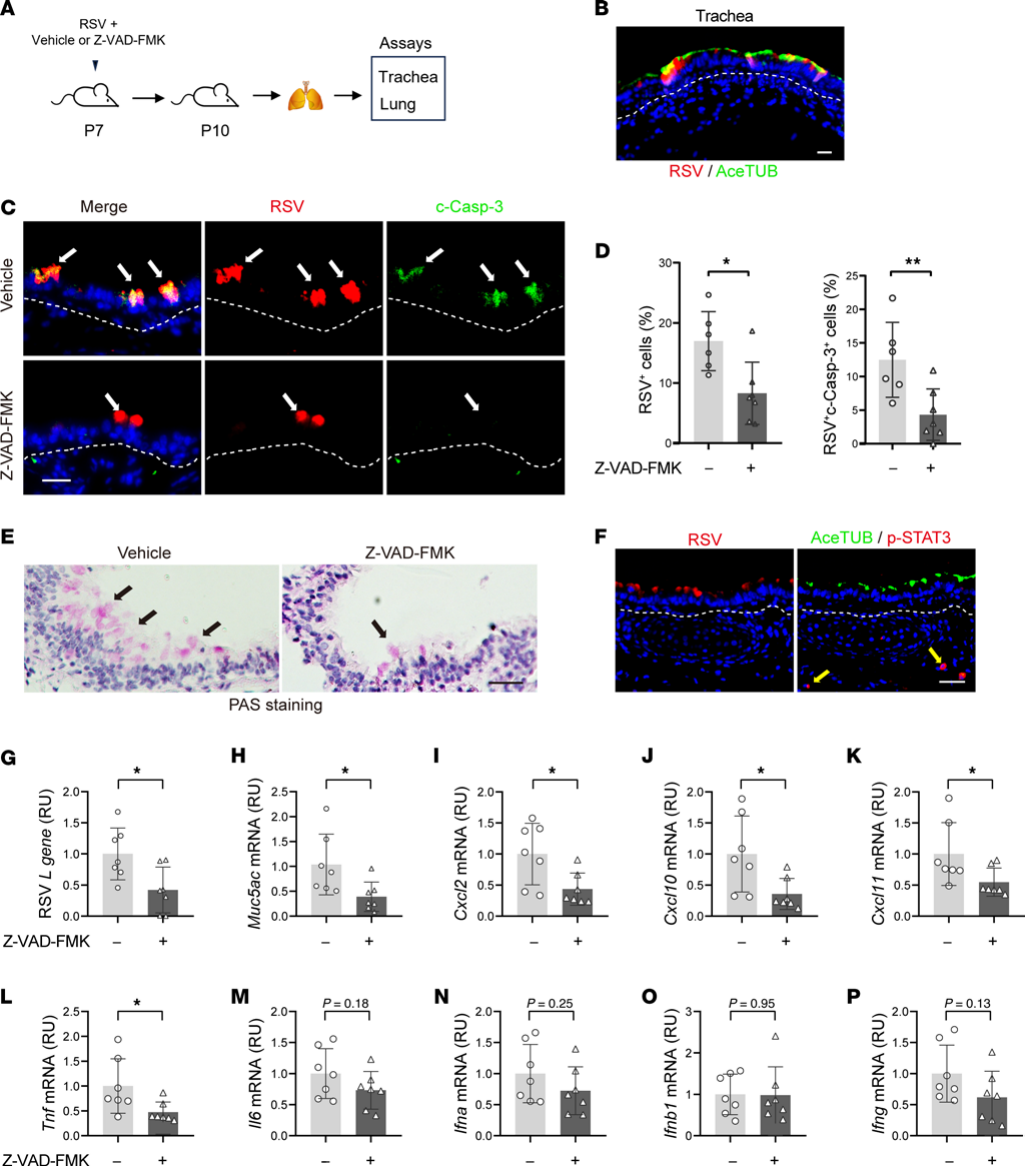
Blockade of apoptosis ameliorates RSV infection and lung inflammation in neonatal mice. (**A**) Schematic of RSV A2-line19F infection with/without Z-VAD-FMK treatment in neonatal BALB/c mice. Neonatal mice were intranasally given with 1 × 10^6^ RSV in 20 μL of Z-VAD-FMK (0.3 mM) or solvent (3% DMSO). The trachea and lung samples were collected at 3 dpi. (**B**) Representative images of RSV (red) and AceTUB (green) staining in mouse trachea. Blue color is nucleus staining and the overlay of red and green shows as yellow color. (**C**) Representative images of RSV (red) and c-Casp-3 (green) staining in control and Z-VAD-FMK–treated mouse trachea. White arrows mark infected cells. (**D**) Relative abundance of RSV ^+^ cells and RSV^+^c-Casp-3^+^ cells in mouse trachea. (**E**) Representative image of periodic acid–Schiff (PAS) staining in trachea sections of neonatal mice. Black arrows mark positive staining. (**F**) Representative images of RSV (red), AceTUB (green), and p-STAT3 (red) staining using 2 adjacent trachea sections (4μm apart) of neonatal mice. Blue color is nucleus staining and the overlay of red and green shows as yellow color. Yellow arrows mark 2 p-STAT3^+^ cells in the parenchyma that are not infected. (**G**–**P**) The relative levels of RSV *L gene* (**G**), *Muc5ac* (**H**), *Cxcl2* (**I**), *Cxcl10* (**J**), *Cxcl11* (**K**), *Tnf* (**L**), *Il6* (**M**), *Ifna* (**N**), *Ifnb1* (**O**), and *Ifng* (**P**) gene expression in neonatal lung at 3 dpi by RT-qPCR. Dotted lines mark basement membrane. Each dot represents 1 mouse. *P < 0.05, **P < 0.01, and ***P < 0.001 by Student’s *t* test. Scale bar: 50 μm.

To demonstrate therapeutic benefits of inhibition of apoptosis in infant RSV disease, we utilized an in vivo model of neonatal BALB/c mice infected with an RSV A2-line19F strain at postnatal day 7 (P7) (27). A caspase inhibitor Z-VAD-FMK was administrated intranasally at the time of infection and mice were analyzed at 3 dpi (Figure 10A). Mixing Z-VAD-FMK with RSV together reduced the volume for 1-time intranasal delivery tolerable to P7 pups and had no effect on RSV infection (Supplemental Figure 14). We evaluated RSV infection and epithelial phenotypes in the trachea, as mouse tracheal epithelium resembles human bronchial epithelium in structure and cellular composition (58), and collected mouse lungs for assessment of inflammation. Like RSV infection in humans, RSV also mainly targeted ciliated cells (Figure 10B). Z-VAD-FMK treatment significantly decreased the abundance of RSV^+^ cells and blocked apoptosis (Figure 10, C and D). In addition, RSV-induced mucus overproduction was ameliorated by Z-VAD-FMK treatment (Figure 10E). Further, we found no significant level of p-STAT3 in RSV-infected ciliated cells in the trachea (Figure 10F). This was not caused by antibody issues, as p-STAT3 was detected in a few tracheal mesenchymal cells that were not infected with RSV (Figure 10F). Negative p-STAT3 staining in infant ciliated cells following RSV infection were also found in the infant hPCLS model (Supplemental Figure 7C). Lastly, Z-VAD-FMK treatment significantly reduced mRNA levels of RSV gene, *Muc5ac*, *Cxcl2/10/11*, and *Tnf-*α (Figure 10, G–L). Other inflammatory genes, including *Il6* and IFN genes, were trending toward a lower level by Z-VAD-FMK (Figure 10, M–P). Importantly, Z-VAD-FMK treatment had no effect on STAT3 phosphorylation (Supplemental Figure 6, F–I). Taken together, blockade of apoptosis ameliorates lung infection and inflammation following RSV infection in vivo.

## Discussion

We employed 2 human bronchial epithelium models — ALI culture and hPCLS — to investigate why infants are susceptible to severe RSV disease. We show that ciliated cells in infants, but not adults, are intrinsically impaired in antiapoptotic STAT3 activation in response to RSV infection. RSV exploits this age-related apoptosis mechanism and subsequent extrusion of RSV-infected apoptotic ciliated cells to facilitate virus spread. Of note, extrusion of apoptotic cells from the epithelium barrier is evolutionarily conserved to protect the barrier function in multiple organs (44, 45). Our results support the hypothesis that sloughing of RSV-infected epithelial cells contributes to spread of infection as well as airway obstruction in severe RSV disease in infants. RSV infection of infant bronchial epithelium induces robust cytokine/chemokine gene expression, which likely serves as an early event to unleash dysregulated inflammatory responses found in severe cases of RSV infection in infants. Supporting this possibility, blockade of apoptosis in a neonatal mouse model of RSV infection reduces viral loads, mucus hyperplasia, and lung inflammation. Our findings indicate that age-related severity of RSV infection in infants is critically regulated at the bronchial epithelium barrier, which provides a new mechanism in addition to previously identified immaturity of the immune system in infants (3, 18, 19). A limitation of our study is that we only had 1 sample from a patient with Job syndrome. To circumvent this limitation, we employed small-molecule inhibitor and shRNA approaches to block STAT3 activities in multiple healthy BSC lines. We also acknowledge that our study lacks bronchial biopsy samples to assess insufficient STAT3 activation during early phases of RSV infection in infants due to technical difficulties. However, this caveat is offset by our findings in the infant hPCLS model of RSV infection. While lung biopsies from fatal cases of infant RSV disease were previously reported (3, 4), bronchial epithelial cell phenotypes in these samples are likely confounded by drug treatment and inflammation to preclude assessment of epithelial responses selectively associated with age.

The bronchial epithelium model derived from TA BSCs reproduces age-related RSV infection in the ex vivo model using hPCLSs prepared from infant and adult donor lungs. Considering that TA BSCs are expanded from a limited number of BSCs in TA samples and passaged multiple times in vitro before differentiation, TA BSCs must retain developmental and age-specific memory that is transmitted to their progeny, including ciliated cells, to mediate age-related responses to RSV. Our transcriptomic analyses by bulk RNA-Seq of BSCs and snRNA-Seq of ALI cultures validate the memory of age on BSCs and the differentiated ciliated cells. Consistent with our findings, a separate study using laser capture–microdissected whole epithelium shows changes in gene expression with age (39). In addition to further characterization of BSCs at different ages, epigenetic and gene expression assays to identify age-related differences in their ciliated cell progeny are also warranted to identify mechanisms that regulate age-related STAT3 activation in ciliated cells following RSV infection. The TA BSC–based human bronchial epithelium model can also be applied to further patients across the age spectrum to investigate the transition from susceptibility to RSV infection during infancy to relative protection in older children and adults and back to susceptibility in the elderly. This approach would allow even deeper understanding of how disease susceptibility is regulated at the RSV-host epithelium interface and whether additional genetic and environmental factors shift this age of transition.

STAT3 is a multifunctional transcription factor that regulates the expression of genes involved in several biological processes such as inflammation, proliferation, and survival (51–54). A variety of cytokines and growth factors, including IL6, IL22, and EGF, can activate STAT3. However, since the presence of adult bronchial epithelial cells in hybrid ALI cultures failed to protect infant ciliated cells from apoptosis following RSV infection, it is unlikely that STAT3 activation in adult ciliated cells is mediated by secreted factors. Instead, STAT3 activation in adult ciliated cells may be activated by RSV interaction with host factors in a cell-autonomous manner. Whether *BCL2* family genes are downstream mediators of STAT3 to regulate apoptotic cell death in RSV-infected ciliated cells warrants future investigation. We show that complete inhibition of apoptosis in the neonatal ALI model fails to completely reduce RSV viral RNA to the low levels found in the adult ALI model. Therefore, in addition to STAT3 regulation of apoptotic cell death, other age-related mechanisms are likely involved in determining severity of epithelial infection by RSV.

RSV is equipped with additional virus-specific factors to capitalize on susceptibility to apoptotic cell death at an early age to cause severe infection. For example, RSV infection induces ciliary dyskinesia and mucus hyperplasia in infants to impair mucociliary clearance (3, 4, 40). RSV nonstructural proteins, including NS1 and NS2, also suppress premature apoptosis to promote viral replication (59). Further, RSV sensitizes infected cells to apoptosis activated by tumor necrosis factor–related apoptosis-inducing ligand (60). These RSV-derived factors can potentially increase the number of apoptotic cells and prolong the time window of virion production and secretion from already-infected and extruded ciliated cells to spread RSV in bronchial epithelium.

In contrast to RSV infection, SARS-CoV-2 infection induces ferroptosis (61) and has no effect on STAT3 activation. We found that SARS-CoV-2 similarly infected neonatal and adult bronchial epithelial cell models. Consistent with our observation, there is no difference in SARS-CoV-2 receptor expression in human lungs between children and adults (62). In addition, a recent publication reported similar SARS-CoV-2 viral replication in human nasal epithelial cells between pediatric (under 12 years) and adult (30–50 years) groups, although the older adult group (over 70 years) appeared to better support SARS-CoV-2 viral infection in nasal epithelium (63). Based on these findings, we conclude that infection of human bronchial epithelial cells with SARS-CoV-2 is not affected by the age of the host until a senior age. The difference in COVID-19 disease severity between adults and children is likely caused by an unchecked inflammatory response in adult patients (64–67) that is different from the immune response in SARS-CoV-2–infected infants and young children (68, 69). The comparison between RSV and SARS-CoV-2 infection of human epithelium models highlights virus-specific mechanisms underlying age-related predilection for severe disease.

In summary, our study identifies impaired antiapoptotic STAT3 activation in ciliated cells following RSV infection as an age-related mechanism underlying severe RSV disease in infants, which can be targeted to reduce infection and inflammation. In addition, the neonatal bronchial epithelium models in ALI culture and hPCLS established in this study can be employed to identify additional molecular mechanisms of age-related RSV infection and to test potential therapeutics.

## Methods

### Sex as a biological variable.

Sex was not considered as a biological variable in this study. Both male and female human samples and mice were used for all studies. The objective of this study was to investigate human bronchial epithelium–derived mechanisms in age-related susceptibility to severe RSV disease in infants. We employed 3 models of RSV infection in this investigation. These include an in vitro human epithelium model generated from neonatal and adult BSCs in ALI, an ex vivo hPCLS model prepared from infant and adult donor lungs, and a neonatal mouse model. Severity of RSV infection was assessed by antibody staining for viral proteins and cell death markers, quantification of viral and host responsive gene expression, and histology. Differences in ciliated cell gene expression at baseline and 1 dpi between the 2 age groups were assessed by snRNA-Seq to identify age-related signaling responses to RSV infection. The role of antiapoptotic STAT3 activation in protection against severe RSV infection was assessed using antagonist and/or agonist treatment approaches in these 3 models.

### Human donor lungs for hPCLSs and histological sections.

Lungs from deidentified and deceased human donors were purchased from the International Institute for the Advancement of Medicine (IIAM, Edison, New Jersey, USA). These donor lungs were declined as transplants and met the criteria of no smoking exposure or any underlying airway diseases. Four adult lungs (1 female and 3 male donors of 43–69 years of age) and 3 lungs from infants (1 female and 2 males of 0–12 months of age) were used in this study. The donors had no previously known lung diseases. Because the experiment involves no intervention or interaction with living individuals, this project is deemed nonhuman subject research by the Institutional Review Board at Massachusetts General Hospital (MGH).

### BSC isolation and expansion.

BSC derivation from TA samples was described previously (30). Briefly, TA samples were collected via in-line suctioning from neonatal patients who were intubated at the Neonatology Intensive Care Unit (NICU) and adult patients at the Neuro-ICU, MGH. Patients were intubated due to cardiovascular and neurogenic respiratory failure. BSCs in TA samples were expanded in Small Airway Epithelial Cell Growth Medium (SAGM, PromoCell, C-21070) in the presence of SMAD/ROCK/mTOR inhibitors (30, 31). BSCs of 1 patient with Job syndrome were isolated from fresh discarded surgical specimens (56) and expanded similarly as TA BSCs.

### RSV preparation.

RSV strain A2 (VR-1540) and RSV strain B (WV/14617/85) (VR-1400) were purchased from ATCC. RSV A2-GFP strain was purchased from ViraTree (https://www.viratree.com/). RSV A2-line19F strain was a gift from Nicholas W Lukacs (University of Michigan, Ann Arbor, Michigan, USA). RSV was propagated in Hep-2 cell line (ATCC CCL-23) as previously described (5). Briefly, Hep-2 cells were cultured in Opti-MEM (Gibco) supplemented with 2% FBS (Gibco) and 1% GlutaMAX Supplement (Gibco). At 80% confluence, Hep-2 cells were infected with RSV in complete medium at a multiplicity of infection (MOI) of 0.1 for 1 hour at 37°C. When high cytopathic effects were observed (approximately 5 dpi) in culture, cells were scraped into the medium, cell debris was separated by centrifugation, and the supernatant was aliquoted and stored at –80°C. RSV virus titers were measured via plaque assay as previously described (70).

### Preparation and RSV infection of hPCLSs.

Human PCLSs were prepared from donor lungs at a thickness of 250 μm and were then cryopreserved using a previously published protocol (71). Frozen hPCLSs were thawed before experimental treatment. For RSV infection, PCLSs were incubated with RSV A2 or RSV A2-GFP viruses (1 × 10^6^ pfu) in the culture media (DMEM/F-12, Thermo Fisher Scientific) supplemented with antibiotic/antimycotic (Thermo Fisher Scientific) for 2 days. PCLSs were then washed with PBS and fixed for 3 hours in 4% PFA, followed by staining and confocal imaging.

### Infection of ALI cultures with RSV.

Frozen aliquots of RSV were thawed and diluted in PBS to a designated titer immediately before use. Prior to infection, the apical surface of ALI cultures was washed 2 times with 200 μL PBS to remove the mucus. RSV suspension (4 × 10^5^ PFU in 100–200 μL) was then applied to the apical surface for 1 hour at 37°C, 5% CO_2_, followed by 3 washes with 200 μL PBS to remove unbound viral particles. ALI cultures were analyzed at multiple time points after infection. The cytopathic effect of RSV-infected ALI cultures was monitored daily by bright field microscopy. Apical washes (200 μL PBS for 20 minutes at 37 °C) were collected to evaluate apoptosis of detached cells and viral spreading from apoptotic cells. At the end of infection experiments, RSV infected ALIs were either fixed for 15 minutes in 4% paraformaldehyde/PBS for IHC or collected in lysis buffer for protein or RNA assays.

### Statistics.

For details on statistical analysis and the number of samples and experimental repeats, see corresponding figure legends and results section. For statistical comparison between 2 experimental groups, unpaired Student’s *t* test was applied. For statistical comparison between more than 2 groups, 1-way ANOVA followed by Tukey’s test or 2-way ANOVA followed by Dunn’s multiple comparison test were statistically appropriate. Statistical tests were performed using GraphPad Prism 8. A calculated *P* value less than 0.05 is considered statistically significant.

### Study approval.

TA samples were collected under an approved IRB protocol (No. 2019P003296). BSCs of 1 patient with Job syndrome were isolated under an approved IRB protocol (No. 2017P001479). RSV infection experiments were performed in the biosafety level 2 (BSL-2) facility. All work with SARS-CoV-2 was performed in the biosafety level 4 (BSL-4) facility of the National Emerging Infectious Diseases Laboratories at Boston University following approved standard operating procedures. All mouse infection procedures were approved by the Institutional Animal Care and Use Committees of Massachusetts General Hospital.

### Data availability.

The raw data of snRNA-Seq and TA BSCs bulk RNA-Seq from this paper is available in the GEO database with the accession number GSE274466, GSE212412, GSE242397, GSE211790, and GSE242397. Values for all data points in graphs are reported in the Supporting Data Values file. All other raw data and materials are available from the corresponding author upon request.

Please see Supplemental Materials for more experimental details.

## Author contributions

CZ performed RSV infection experiments and analyzed the snRNA-seq data. YB prepared hPCLSs and assisted the assay of RSV infection of hPCLSs and mouse models. WW analyzed the transcriptomes of neonatal and adult BSCs. GMA derived TA BSCs. HM provided BSCs from a patient with Job syndrome. JO, AJH, and EM performed SARS-CoV-2 infection experiment. NWL provided RSV A2-line19F strain. RF guided RSV infection experiments. XA performed antibody staining of human lung samples. XA and PHL conceived the study. CZ and XA wrote the manuscript. PHL edited the manuscript. All the authors read and commented on the manuscript.

## Supplementary Material

Supplemental data

Unedited blot and gel images

Supporting data values

## Version 1 11/01/2024

Electronic publication
