# Supplementary material for: Activation of STAT3-mediated ciliated cell survival protects against severe infection by respiratory syncytial virus
Source: J Clin Invest. 2024 Nov 1;134(21):e183978. doi: 10.1172/JCI183978 (PMC11527452; doi:10.1172/JCI183978)
Supplement: Supplemental data [file jci-134-183978-s012.pdf]

SUPPLEMENTARY INFORMATION

Supplementary materials and methods

Transcriptome Analysis of BSCs by bulk RNA-seq

Bulk RNA-seq service was provided by Active Motif (Carlsbad, CA, USA). As previously described (1), RNA (2 µg per sample) extracted from $1x10^6$ BSCs was performed using the Qiagen RNA RNeasy Kit (Cat#74104, Qiagen) followed by library preparation with the Illumina TruSeq V2 Kit (Cat#20020594, Illumina). Libraries were sequenced on Illumina NextSeq 500 as paired-end 42-nt reads and the reads were subsequently mapped to the human hg38 reference genome using STAR algorithm version 2.6.0a. Only genes with an average count above 2 were analyzed. The DESeq2 R package (1.30.0) was used to perform differential expression and principal component analyses. Genes with an adjusted p value less than 0.05 (padj<0.05) were considered as differentially expressed genes (DEGs).

SARS-CoV-2 preparation

SARS-CoV-2 stocks [isolate USA_WA1/2020, provided Dr. Natalie Thornburg at CDC and the World Reference Center for Emerging Viruses and Arboviruses (WRCEVA)] were propagated in Vero E6 cells (ATCC CRL-1586) cultured in Dulbecco's modified Eagle's medium (DMEM) supplemented with 2% FBS, penicillin (50 U/mL), and streptomycin (50 mg/mL). To remove confounding cytokines and other factors, viral stocks were purified by ultracentrifugation through a 20% sucrose cushion at 80,000 g for 2 h at 4°C as previously described (2). SARS-CoV-2 titers were determined in Vero E6 cells by tissue culture infectious dose 50 (TCID50) assay.

Infection of ALI cultures with SARS-CoV-2

Frozen aliquots of SARS-CoV-2 were thawed and diluted in PBS to a designated titer immediately before use. Prior to infection, the apical surface of ALI cultures was washed 2 times with 200 µL PBS to remove the mucus. SARS-CoV-2 suspension was then applied to the apical surface for 1 h at 37°C, 5% CO_2, followed by 3 washes with 200 µL PBS to remove unbound viral particles. ALI cultures were analyzed at multiple time points after infection. Following approved inactivation procedures, SARS-CoV-2 infected cells were fixed for at least 6 hours with 4% paraformaldehyde and removed from the maximum containment laboratory for further analysis.

Preparation and analysis of single nuclei RNA-seq (snRNA-seq) of ALI cultures

To overcome the technical issue in isolating live ciliated cells, we optimized a protocol of treatment of day 21 ALI cultures with Accutase (Cat#A1110501, Thermo Fisher Scientific). For one 24-well insert, the membrane was removed from the insert and treated with 500 μl of Accutase with EDTA and EGTA (5 mM each) for 30 minutes at 37℃ on a sharker. After treatment, cells were dissociated by titrating and cell pellets were collected by centrifugation. After 1X PBS wash to remove the remaining Accutase, cells were resuspended in nuclear extraction buffer according to manufacturer's instruction (10X Genomics, protocol CG000124). The sequencing service of snRNA-seq was provided by Whitehead genome core of MIT (Boston, MA, USA). The RNA libraries were sequenced on Illumina NovaSeqS4 (150x150 Paired-End). The Cell Ranger output files were provided by the Whitehead genome core. The Seurat R package (5.0.1) was used to perform differential expression and cluster-finding analyses of snRNA-seq data. We removed doublets and nuclei exhibiting fewer than 1,250 genes or >30% of mapped reads originating from the mitochondrial genome. The step of "FindClusters" for all nuclei was performed at a resolution of 0.1. The GSEA of ciliated cells was performed by the Singleseqgset R package (0.1.2.9000). Genes with an adjusted p value less than 0.05 (padj<0.05) were considered as DEGs.

Mucociliary differentiation of BSCs in ALI

As previously described (3), $2x10^5$ BSCs were seeded onto the 6.5 mm Transwell with 0.4 μm Pore Polyester Membrane Insert (Cat# 3470, Corning) that was precoated with 804G-conditioned medium. Differentiation in PneumaCult medium (Cat# 05001, StemCell Technology) started after removal of medium from the top chamber and completed after 21 days in ALI. Medium was changed every 2 days. At day 21 in ALI, cultures were processed for antibody staining and viral infection assay. For hybrid ALI culture experiments, neonatal BSCs were infected with a GFP lentivirus and adult BSCs were infected with a empty lentivirus followed by treatment with puromycin (1 μg/mL) for 3 days to select for GFP-transduced BSCs. GFP^+ neonatal BSCs were then mixed with adult BSCs at a 1:1 ratio and subjected to differentiation in ALI. For Dox-inducible STAT3 knockdown experiments in ALI cultures, after screening with puromycin (1 μg/mL) for 3 days, BSCs were subjected to differentiation in ALI culture. At 18 days of ALI differentiation, the cultures were treated with vehicle or Dox (500 ng/ml) in the basal medium. The knockdown efficiency and infection were performed at 21 days of differentiation. Lentivirus was generated from 293T cells using Lipofectamine 3000 Transfection Reagent (Cat#L3000015, Thermo Scientific).

Infection of neonatal mouse

BALB/c mice were purchased from Charles River Laboratories, USA. All mice were bred under specific

pathogen-free conditions. The protocols were approved by the Institutional Animal Care and Use Committee at Massachusetts General Hospital (2019N000137, Principal Investigator: X.A). 7-day old (P7) neonatal mice were infected with 1×10^6 RSV A2-line19F viruses. 3% DMSO or Z-VAD-FMK (10 mM) was diluted in the viral inoculum and intranasally given to the neonatal mice. Trachea and lung samples of infected mice were collected at 3 dpi to perform histology staining and measure gene expression.

Antibody staining of ALI cultures and quantification

Mock or virus-infected ALI cultures were fixed for 15 min (RSV) or at least 6 hours (SARS-CoV-2) in 4% paraformaldehyde/PBS before they were processed for cryosectioning at a thickness of 10 µm or whole-mount staining. Sections or whole-mount ALI cultures were immune-stained according to a standard protocol using primary antibodies and secondary antibodies listed in the antibody table. To identify different epithelial cell types in ALI cultures, sections were stained for ciliated cells (rabbit anti-RFX3, 1:100, Cat# HPA035689,Sigma-Aldrich, and mouse anti-AceTUB, 1:100, Cat# T6793, Sigma-Aldrich), club cells (rabbit anti-SCGB1A1, 1:100, Cat# HPA031828, Sigma-Aldrich), goblet cells (mouse anti-MUC5AC, 1:100, Cat# MA5-12178, Thermo Fisher Scientific), and BSCs (rabbit anti-TP63, 1:100, Cat# 13109, Cell Signaling Technology). Additional antibodies used for the infected ALI cultures include: mouse anti-RSV Fusion protein (1:100, Cat# MCA490, Bio-Rad), rabbit anti-Cleaved Caspase-3 (Asp175) (1:100, Cat# 9661, Cell Signaling Technology), rabbit anti-phospho-RIP (Ser166) (D8I3A) (1:100, Cat# 44590S, Cell Signaling Technology), rabbit anti-SARS-CoV nucleocapsid protein (N) antibody that cross-reacts with SARS-CoV-2 N (1:2000, Cat# 200-401-A50, Rockland Immunochemicals), rabbit anti-ZO1(1:100, Cat# ab216880, Abcam), and rabbit Phospho-Stat3 (Tyr705) (1:25, Cat# 9145, Cell Signaling Technology). Nuclei were stained with Hoechst dye (1:500, ThermoFisher, Cat# H3570). The following secondary antibodies were used accordingly: goat anti-rabbit Alexa Fluor 594 (IgG, 1:200, Cat#A-11037, Thermo Scientific) and goat anti-mouse Alexa Fluor 488 (IgG, 1:200, Cat#A-11029, Thermo Scientific).

For quantification, 4-6 randomly selected and non-overlapping fields in cross sections collected from ALI cultures of one BSC lines in 2-3 independent experiments were imaged at 20X. The total number of epithelial cells per image was counted based on the nuclei countered stained with Hoechst dye. More than 1000 nuclei were quantified for each ALI culture. Epithelial cells labelled with specific markers in the same image were counted and reported as the percentage of the epithelial cell population.

Quantitative Real Time PCR (qPCR)

Total RNA of ALI culture was extracted using a RNeasy kit (Qiagen, 74106) and total RNA of mouse lung was extracted using TRIzol™ Reagent (Invitrogen, 15596026) followed by reverse transcription using Superscript III Reverse Transcriptase (Thermo Fisher Scientific, 18080-044). Real time PCR was performed with SYBR Green Mix (Cat#4367659, Thermo Scientific) using CFX96 real-time system (Bio-Rad). Relative gene expression was measured by normalizing to *GAPDH* using ΔΔCt (cycle threshold difference). All primers were listed in Table S2.

Western Blot

ALI protein was harvested in 1X cell lysis buffer (Cat#9803S, Cell Signaling Technology) with protease inhibitor (Cat#11697698001, Sigma-Aldrich) and phosphatase inhibitor cocktails (Cat#524627-1EA, EMD Millipore) followed by standard Western blot assays. Primary antibodies include rabbit anti-phosphorylated STAT3 at Tyr705 (1:1000, Cat#9145S, Cell Signaling Technology), rabbit anti-STAT3 (1:1000, Cat#12640S, Cell Signaling Technology), rabbit anti-Cleaved Caspase-3 (Asp175) (1:1000, Cat#9661S, Cell Signaling Technology), and mouse anti-β-actin (1:2000, Cat#A5441, Sigma Aldrich). HRP-conjugated secondary antibodies include goat anti-rabbit (1:3000; Santa Cruz Biotechnology, sc-2004) and goat anti-mouse (1:3000; BD Biosciences, 554002). The antigen-antibody complex was detected by SuperSignal™ West Dura Extended Duration Chemiluminescent Substrate (Cat#34075, Thermo Fisher).

Immunohistochemistry of human lung and mouse trachea tissues

Paraffin sections (4 μm) of human lung and mouse trachea samples were processed for immunostaining according to standard protocols. Primary antibodies for human samples include rabbit anti-RFX3 (1:100, Cat#HPA035689, Sigma Aldrich), rabbit anti-KRT5 (1:100, Cat# ab52635, Abcam), rabbit anti-Cleaved Caspase-3 (Asp175) (1:100, Cat# 9661, Cell Signaling Technology), and rabbit anti-SARS-CoV nucleocapsid protein (N) antibody that cross-reacts with SARS-CoV-2 N (1:2000, Cat# 200-401-A50, Rockland Immunochemicals). Primary antibodies for mouse samples include rabbit anti-RSV polyclonal antibody (1:500, Cat# 10286-920, Bioss), rabbit anti-Cleaved Caspase-3 (Asp175) AF488-conjugated (1:100, Cat# 9603, Cell Signaling Technology), rabbit anti-STAT3 (1:50, Cat#12640S, Cell Signaling Technology). Biotinylated goat anti-rabbit (IgG, 1:200, Cat#BA-1000, Vector Laboratories) or goat anti-rabbit Alexa Fluor 594 (IgG, 1:200, Cat#A-11037, Thermo Scientific) were used as the secondary antibody. PAS staining of mouse trachea was done using PAS Staining Kit (Sigma-Aldrich, Cat#395B-1KT). Alcian blue staining was done with alcian-blue solution (Millipore, Cat# TMS-010-C). For DAB staining, sections were treated with standard ABC kit (Vector Labs, PK-6100) and DAB Peroxidase Substrate Kit (Vector

Labs, SK-4100). After DAB staining, samples were counterstained with Hematoxylin QS (Vector Laboratories, H-3404). Bright field images were taken using a digital camera (Nikon DS-Fi2).

Imaging and quantification

Stained slides were examined with a Nikon Ti inverted fluorescence/confocal microscope or Zeiss AX10 brightfield microscope. All images were processed using ImageJ software. Quantification was performed by two independent and blinded examiners as follows. For ALI, trachea, and lung sections, 4-6 individual 20X images per sample were randomly selected and captured. The number of epithelial cells per image was counted based on the nuclei countered stained with DAPI. Epithelial cells labelled with lineage-specific markers or RSV F protein in the same image were counted and reported as the percentage of the epithelial cell population of interest.

Supplementary Table 1. Demographic information of patients from whom TA BSCs were derived.

**Neonatal BSC lines (patients being treated at NICU)**					RSV$^+$ cells (2 dpi)
Patient Study ID	Gestational Age (weeks)	Sex	Disease History	Assays performed	
N1	39	M	NAS	RS, DI, RI	32%
N2	41	F	HIE	RS, DI, RI, SI	22%
N3	38	F	Fetal maternal hemorrhage, polyhydramnios, PPHN	RS, DI	
N4	41	M	PPHN, HIE	RS, DI, RI	19%
N5	40	M	Pneumothorax	RS, DI, RI, SI	20%
N6	41	M	Amniotic fluid aspiration, PNA	RS, DI, RI	24%
N7	39	F	PPHN	RS, DI	
N8	40	M	PPHN, pulmonary hemorrhage	RS, DI	
N9	39	F	Feeding problem	DI, RI, snRNA-seq	30%
N10	37	M	Feeding problem, seizures	DI, RI	28%
**Adult BSC lines (patients being treated in neuro-ICU)**					
Patient Study ID	Age (years)	Sex	Disease History	Assays performed	
A1	36	F	No known respiratory disease	RS, DI, RI, SI, snRNA-seq	4%
A2	37	M	Former smoker	RS, DI, RI	3%
A3	32	M	Former smoker	RS, DI	
A4	45	M	Former smoker	RS, DI, RI	2%
A5	55	F	No known respiratory disease	DI, RI, SI	6%

***NAS:** neonatal abstinence syndrome, **HIE:** hypoxic-ischemic encephalopathy, **PPHN:** persistent pulmonary hypertension of the newborn, **PNA:** pulmonary nodular amyloidosis

***RS** = RNA Sequencing, **DI** = Differentiation, **RI** = RSV infection, **SI** = SARS-CoV-2 infection

Supplementary Table 2. List of primers used for RT-qPCR.

Gene	Forward Primer	Reverse Primer
RSV L2	GAACTCAGTGTAGGTAGAATGTTTGCA	TTCAGCTATCATTTTCTCTGCCAAT
Human *CXCL10*	GAAATTATTCCTGCAAGCCAATTT	TCACCCTTCTTTTTCATTGTAGCA
Human *BCL2*	GGTGGGGTCATGTGTGTGG	CGGTTCAGGTACTCAGTCATCC
Human *MCL1*	GTGCCTTTGTGGCTAAACACT	AGTCCCGTTTTGTCCTTACGA
Human BIRC5	AGGACCACCGCATCTCTACAT	AAGTCTGGCTCGTTCTCAGTG
Human *BCL2L1*	GAGCTGGTGGTTGACTTTCTC	TCCATCTCCGATTCAGTCCCT
Human *IFNL1*	AATTGGGACCTGAGGCTTCTC	CCAGCGGACTCCTTTTTGG
Human *IFNL3*	TAAGAGGGCCAAAGATGCCTT	CTGGTCCAAGACATCCCCC
Human *GAPDH*	GGAGCGAGATCCCTCCAAAAT	GGCTGTTGTCATACTTCTCATGG
Human *TNFA*	GAGGCCAAGCCCTGGTATG	CGGGCCGATTGATCTCAGC
Human *IFNB*	ATGACCAACAAGTGTCTCCTCC	GGAATCCAAGCAAGTTGTAGCTC
Human *MUC5AC*	CTCCTACCAATGCTCTGTA	GTTGCAGAAGCAGGTTTG
Human *MUC5B*	GACAGAGACGACAATGAG	CCTGATGTTTTCAAAAGTTTC
Mouse *18s*	CCATTCGAACGTCTGCCCTAT	GTCACCCGTGGTCACCATG
Mouse *Cxcl2*	CCAACCACCAGGCTACAGG	GCGTCACACTCAAGCTCTG
Mouse *Cxcl10*	CCGGAATCTAAGACCATCAAG	GAGGCTCTCTGCTGTCCATC
Mouse *Cxcl11*	GGGCCGATGCAAAGACA	GAGATGAACAGGAAGGTCACAG
Mouse *Il6*	GGCCTTCCCTACTTCACAAG	ATTTCCACGATTTCCCAGAG
Mouse *Tnf*	CCCTCACACTCAGATCATCTTCT	GCTACGACGTGGGCTACAG
Mouse *Ifna*	CTTCCACAGGATCACTGTGTACCT	TTCTGCTCTGACCACCTCCC
Mouse *Ifnb*	AGATCAACCTCACCTACAGG	TCAGAAACACTGTCTGCTGG
Mouse *Ifng*	ATGAACGCTACACACTGCATC	CCATCCTTTTGCCAGTTCCTC
Mouse Muc5ac	TaqMan primer (Mm01276704_m1) purchased from Thermo Fisher Scientific	
**shRNA target sequence**		
STAT3 shRNA	GGCGTCCAGTTCACTACTAAA	

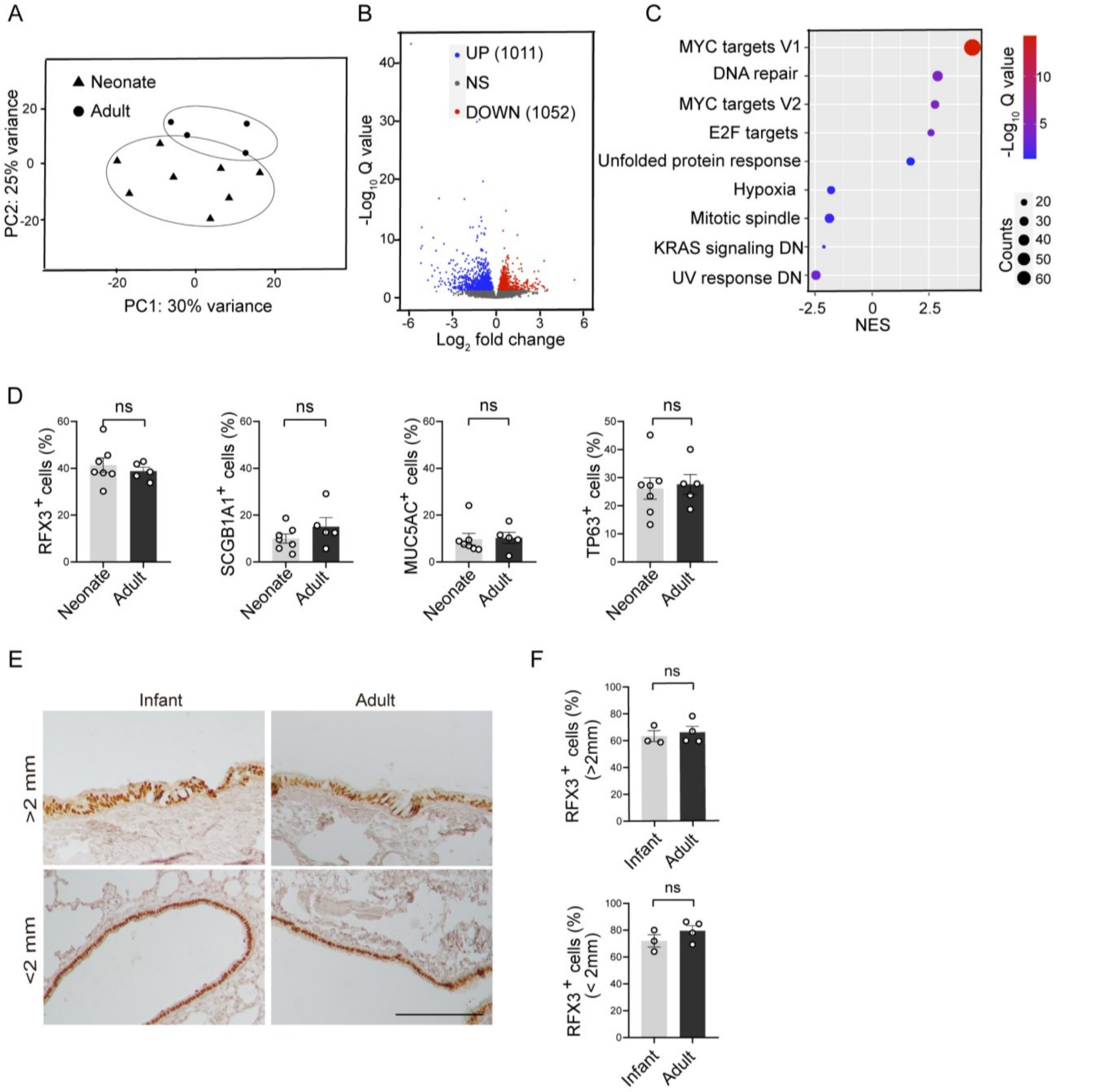

Figure S1. BSCs from infants and adults have similar differentiation potentials. Related to Figure 1.

(A) Principal component (PC) analysis of bulk RNA sequencing datasets of TA BSC lines from neonates and adults. (B) Volcano plot showing the number of differentially expressed genes in neonatal BSCs compared to adult BSCs with an adjusted p value <0.05. (C) Pathway analysis of bulk RNA sequencing results of neonatal and adult TA BSCs. (D) The relative abundance of different epithelial cell types based on antibody staining of day 21 ALI cultures of neonatal and adult TA BSCs. (E) Representative RFX3 staining of healthy human donor lungs from infants and adults. Scale bar, 200 µm. (F) The relative abundance of $RFX3^{+}$ ciliated cells in human donor lungs of infants and adults. Each dot represents one donor. Bar graphs show mean ± SEM. ns, not significant by Student’s t-test (two-tailed) (D and F).

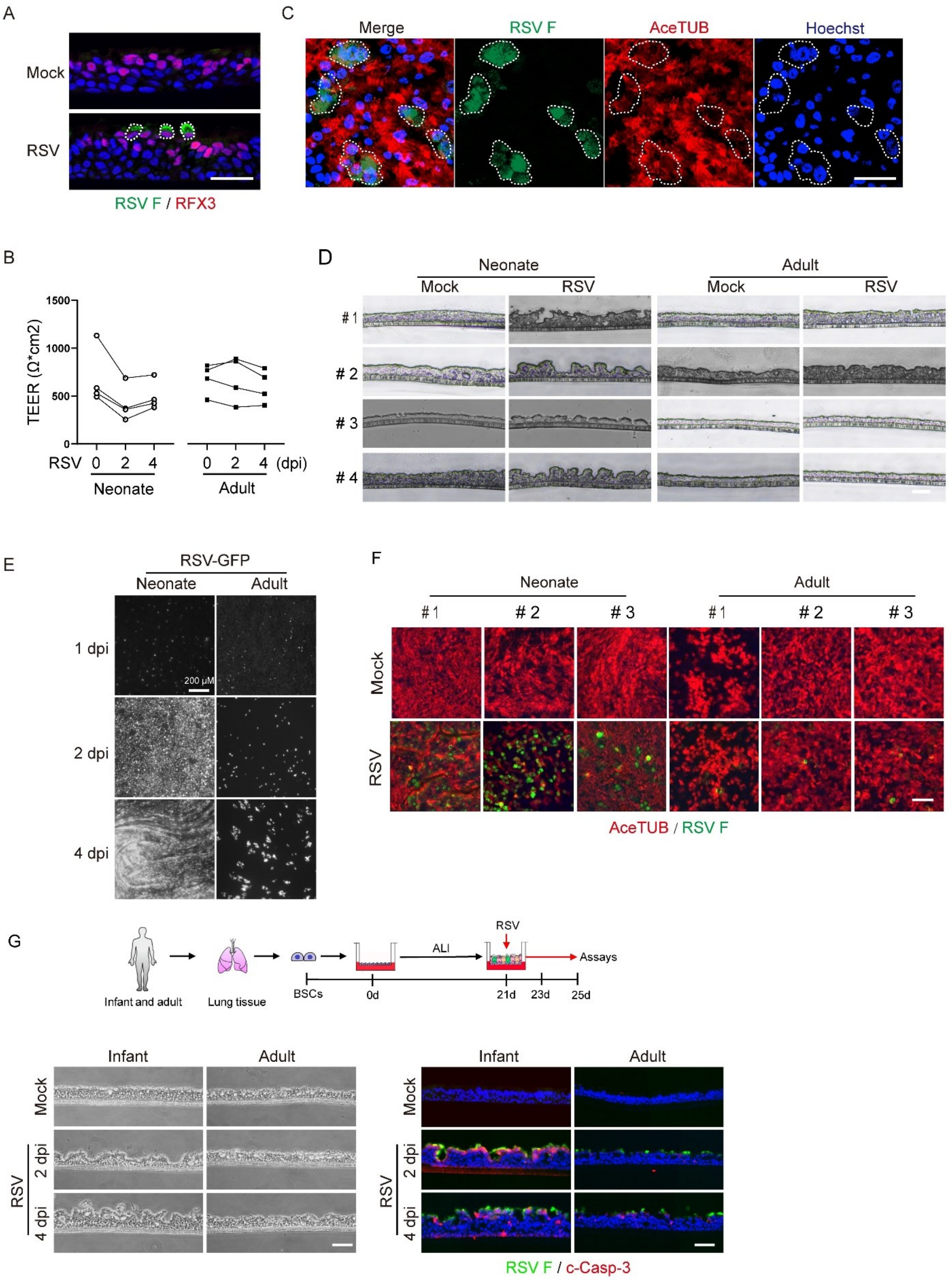

Figure S2. Neonatal and adult ALI cultures show age-related severity of RSV infection. **Related to Figure 1 and 2.**

(A) Representative images of double staining for RFX3 and RSV F protein in ALI cultures at 2 dpi. (B) TEER measurements (Ω*cm2) prior to infection (0 dpi) and at 2 and 4 dpi. (C) Representative whole-mount staining for AceTUB and RSV F protein in adult ALI cultures at 2 dpi. Ciliated cells infected with

RSV were outlined. (D) Brightfield images of ALI cultures from 4 donors in each age group with and without RSV infection at 2 dpi. (E) Representative images of neonatal and adult ALI cultures with and without RSV-GFP infection at 1, 2, and 4 dpi. (F) Representative whole-mount staining for AceTUB and RSV F protein in ALI cultures of 3 donors in each age group with and without RSV infection at 2 dpi. (G) RSV infection of ALI cultures generated with BSCs isolated from biopsy samples of infant (n=2 donors) and adult donor lungs (n=4 donors).

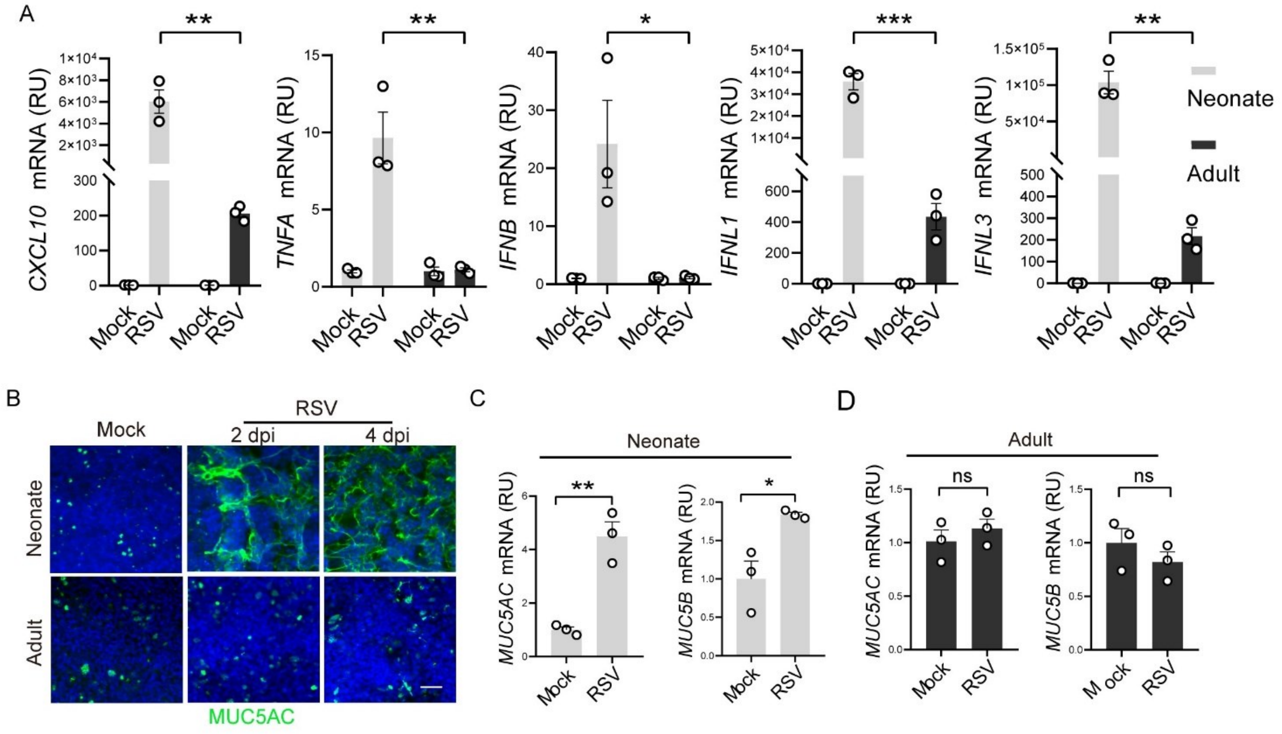

Figure S3. RSV infection elevates more robust cytokine/chemokine and mucin gene expression in neonatal ALI cultures than adult ALI cultures at 2 dpi. Related to Figure 1.

(A) The relative levels of *CXCL10*, *TNFA*, *IFNB*, *IFNL1*, and *IFNL3* gene expression in neonatal and adult ALI cultures at 2 dpi by RT-qPCR. (B) Representative whole-mount staining for MUC5AC. (C and D) The relative levels of *MUC5AC* and *MUC5B* gene expression in neonatal and adult ALI cultures at 2 dpi by RT-qPCR. Each dot represents one donor. Bar graphs show mean ± SEM. *p<0.05, **p<0.01, and ***p<0.001 calculated by two-way ANOVA followed by Dunn's test in (A) and Student's t-test (two-tailed) in (C and D).

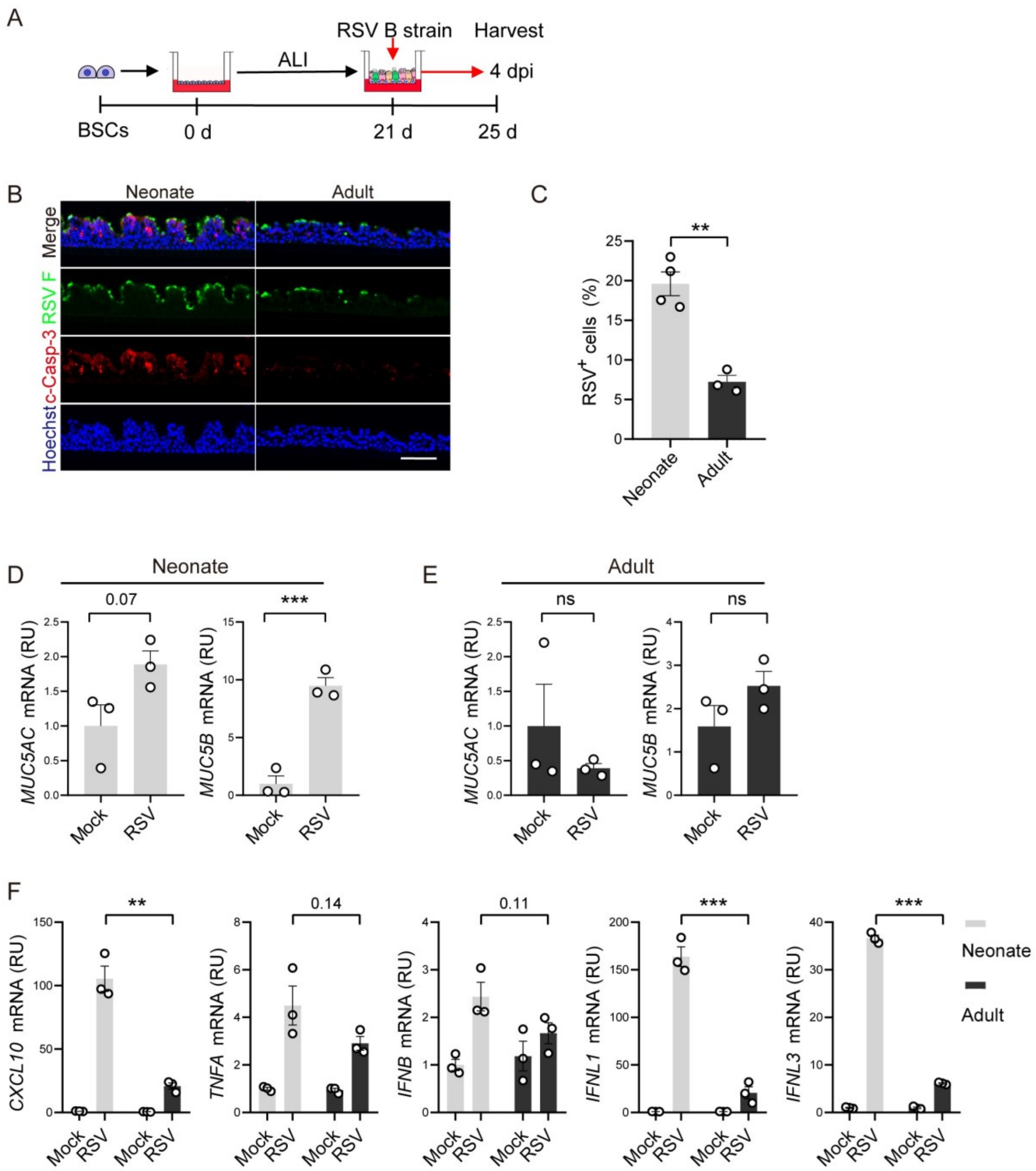

Figure S4. RSV strain B induces age-related severity of infection in neonatal bronchial epithelium model. Related to Figures 1 and 2.

(A) Schematic of infection of neonatal and adult ALI cultures with RSV B virus (WV/14617/85, MOI 2). Assays were performed at 4 dpi. (B) Representative double staining for RSV F protein and c-Casp-3. (C) The relative abundance of RSV F$^+$ cells. (D-F) The relative mRNA levels of *MUC5AC*, *MUC5B, CXCL10*, *TNFA*, *IFNB*, *IFNL1*, and *IFNL3* by RT-qPCR.

Each dot represents one donor. Bar graphs show mean ± SEM. *p<0.05, **p<0.01, and ***p<0.001 calculated by Student's t-test (two-tailed) in (C-E) and two-way ANOVA followed by Dunn's test in (F).

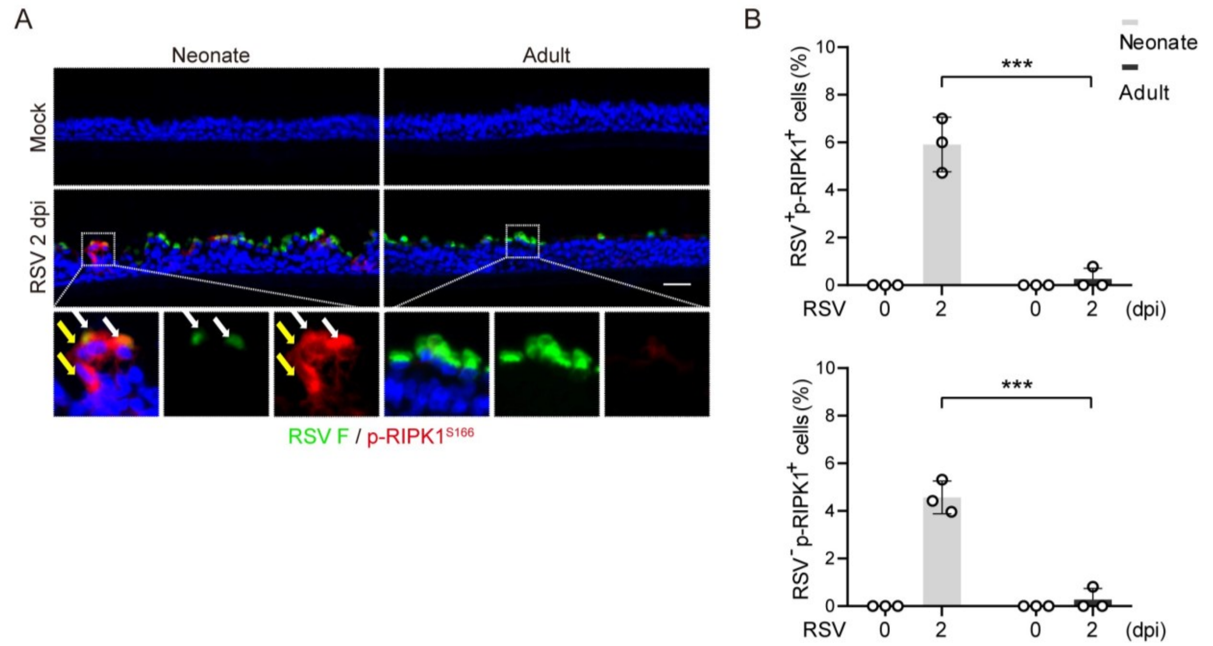

Figure S5. RSV infection induces necroptosis in a small number of epithelial cells in neonatal ALI cultures. Related to Figure 2.

(A) Representative double staining for RSV F protein and p-RIPK1^{S166} using sections of neonatal and adult ALI cultures at 2 dpi. White arrows mark RSV F^{+}p-RIPK1^{+} cells and yellow arrows mark RSV F^{-}p-RIPK1^{+} cells.

(B) Relative abundance of RSV F^{+}p-RIPK1^{+} and RSV F^{-}p-RIPK1^{+} cells.

Each dot represents one donor. Bar graphs represent mean ± SEM. Statistical significance was calculated by two-way ANOVA followed by Dunn's test in (B). ***p<0.001. Scale bar, 50 µm.

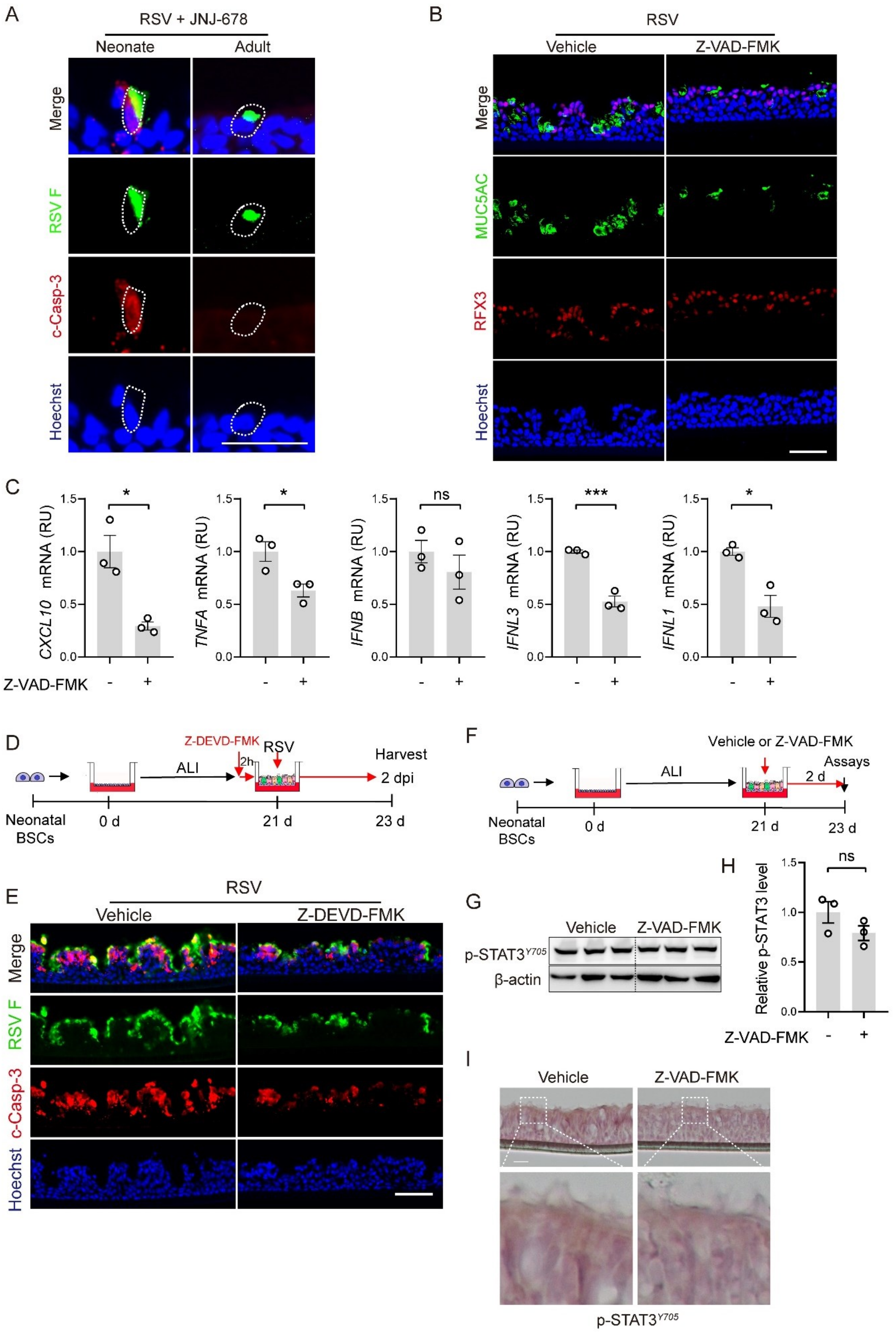

Figure S6. Blockade of apoptosis in neonatal bronchial epithelium following RSV infection reduces mucus hyperplasia and cytokine/chemokine gene expression. Related to Figures 2, 3 and 10.

(A) Representative double staining for RSV F protein and c-Casp-3 in JNJ-678-treated neonatal ALI

cultures at 2 dpi. JNJ-678 treatment was described in Figure 1J. (B) Representative double staining for MUC5AC and RFX3 in neonatal ALI cultures treated with Z-VAD-FMK at 2 dpi. Z-VAD-FMK treatment was described in Figure 2E. (C) Relative levels of *CXCL10*, *TNFA*, *IFNB*, *IFNL3*, and *IFNL1* expression with and without Z-VAD-FMK treatment at 2 dpi by RT-q-PCR. (D) Schematic of Z-DEVD-FMK (40 μM) treatment. (E) Representative double staining for RSV F protein and c-Casp-3 with and without Z-DEVD-FMK treatment at 2 dpi. (F) Schematic of Z-VAD-FMK (40 μM) treatment to test the effect on STAT3 activation. (G) Western blot analyses of p-STAT3^{Y705} levels. β-Actin was loading control. (H) Densitometry measurements of p-STAT3^{Y705} levels normalized to β-actin. (I) Representative DAB staining for p-STAT3^{Y705}. Scale bar, 50 μm. Each dot represents donor. Bar graphs show mean ± SEM. *p<0.05 and ***p<0.001 calculated by Student's t-test (two-tailed). Scale bars, 50 μm.

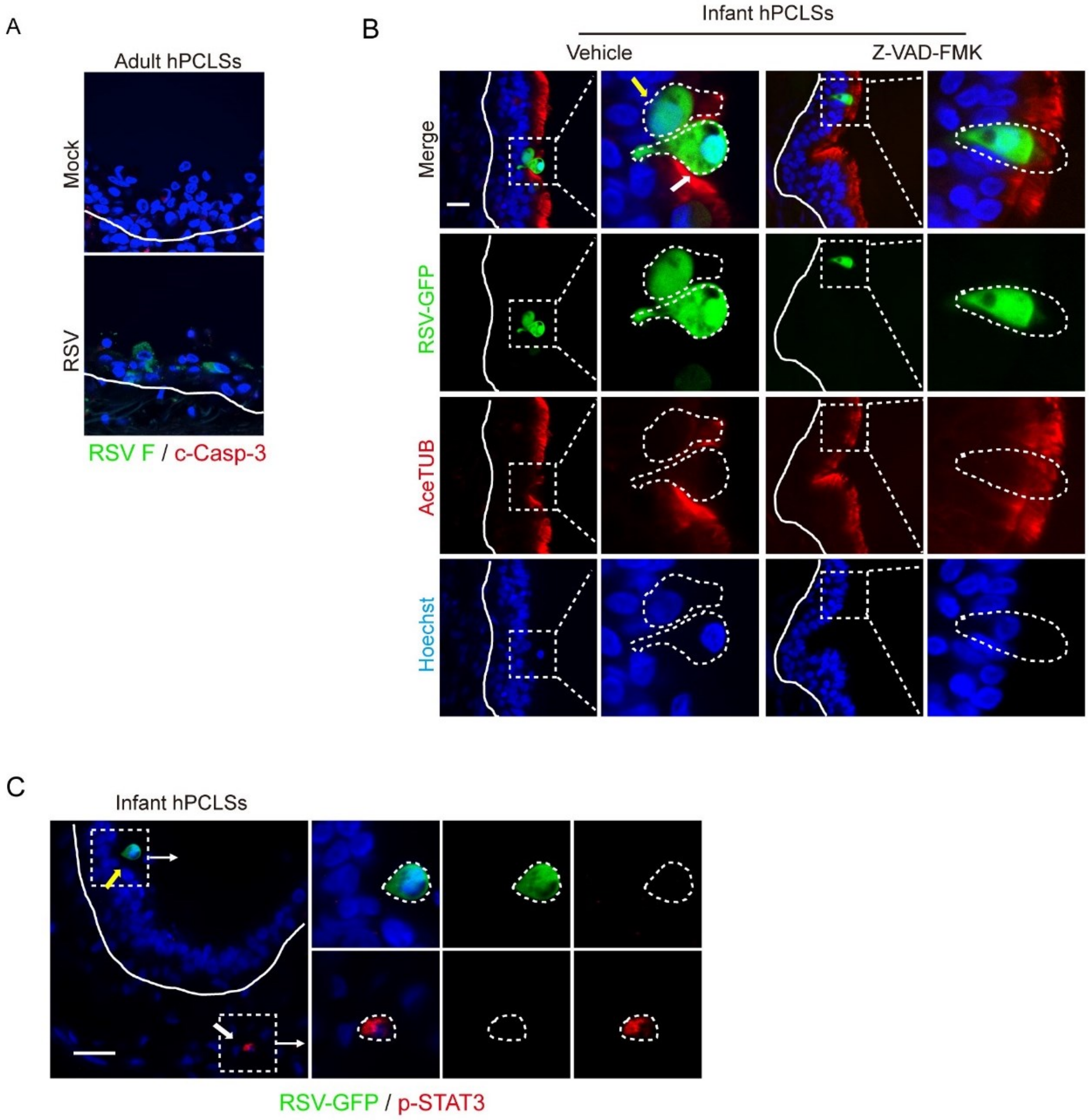

Figure S7. Blockage of apoptosis reduces RSV-induced cell extrusion in infant hPCLSs. Related to Figures 3, 4 and 10.

(A) Representative double staining for RSV F protein and c-Casp-3 in adult hPCLSs at 2 dpi (n=3 donors).

(B) Representative images of RSV-GFP and AceTUB staining in control and Z-VAD-FMK-treated infant hPCLSs. The contour of RSV-GFP-infected ciliated cells is outlined in panels showing enlarged areas. White arrow marks an RSV-GFP$^+$ ciliated cell with complete cilia loss. Yellow arrow marks an RSV-GFP$^+$ ciliated cell with partial cilia loss.

(C) Representative images of RSV-GFP and p-STAT3 staining in RSV-infected infant hPCLSs. Yellow arrow marks an RSV-GFP$^+$ ciliated cell. White arrow marks a p-STAT3$^+$ cell in lung parenchyma that are not infected.

Solid lines mark basement membrane. Scale bar, 50 μm.

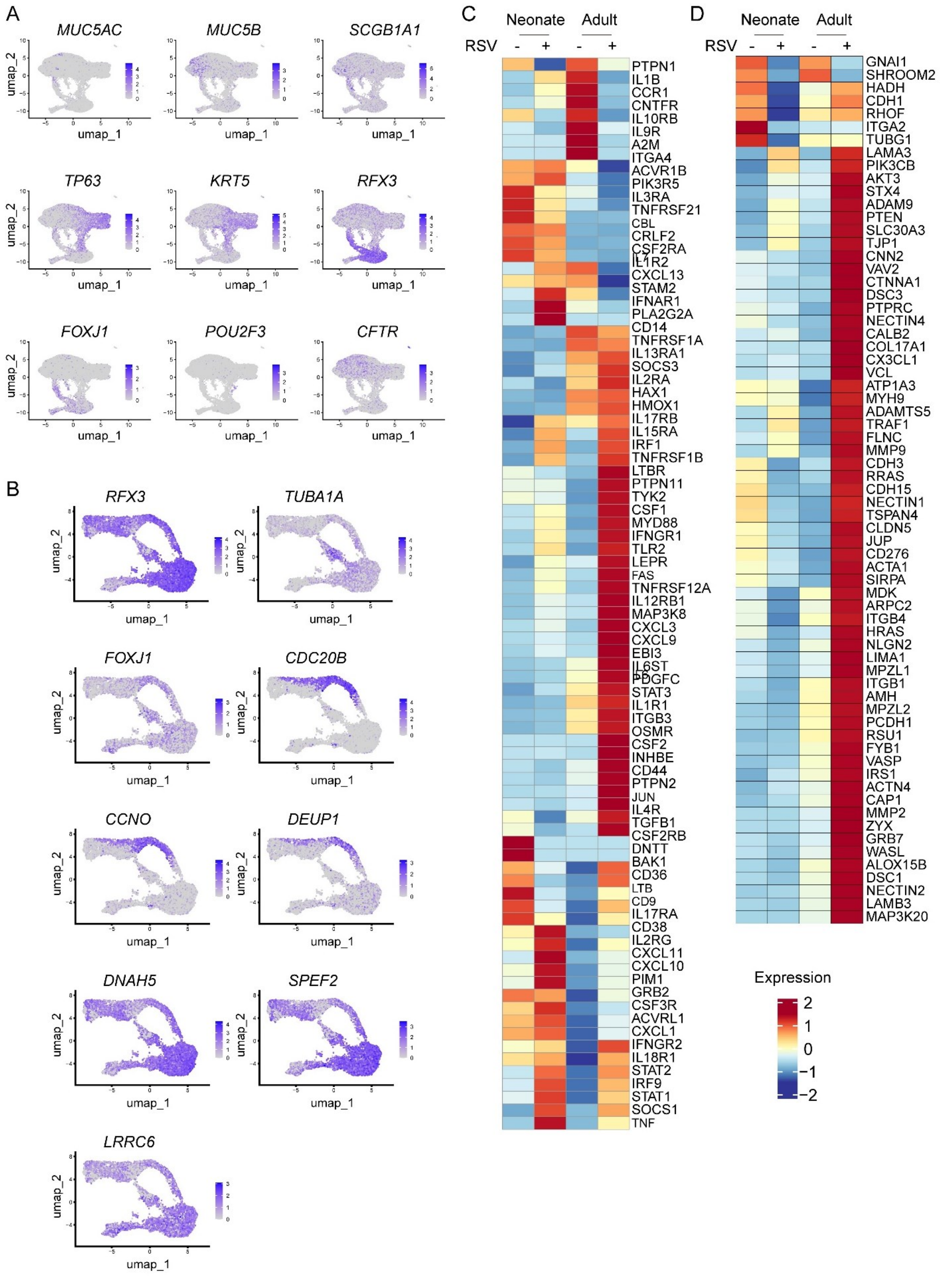

Figure S8. Adult ciliated cells show significant changes in expression of anti-apoptosis and apical junction-related genes in response to RSV infection at 1 dpi. Related to Figure 6.

(A) Feature plots showing epithelial cell markers in different cell clusters. (B) Feature plots showing expression of *RFX3*, TUBA1A, *FOXJ1*, *CDC20B*, *CCNO*, *DEUP1*, *DNAH5*, *SPEF2*, and *LRRC6* genes in all ciliated cells. (C and D) Heatmaps showing relative expression of genes in the IL6_ JAK_ STAT3_SIGNALING (C) and APICAL JUNCTION (D) pathways in neonatal and adult ciliated cells with and without RSV infection at 1 dpi.

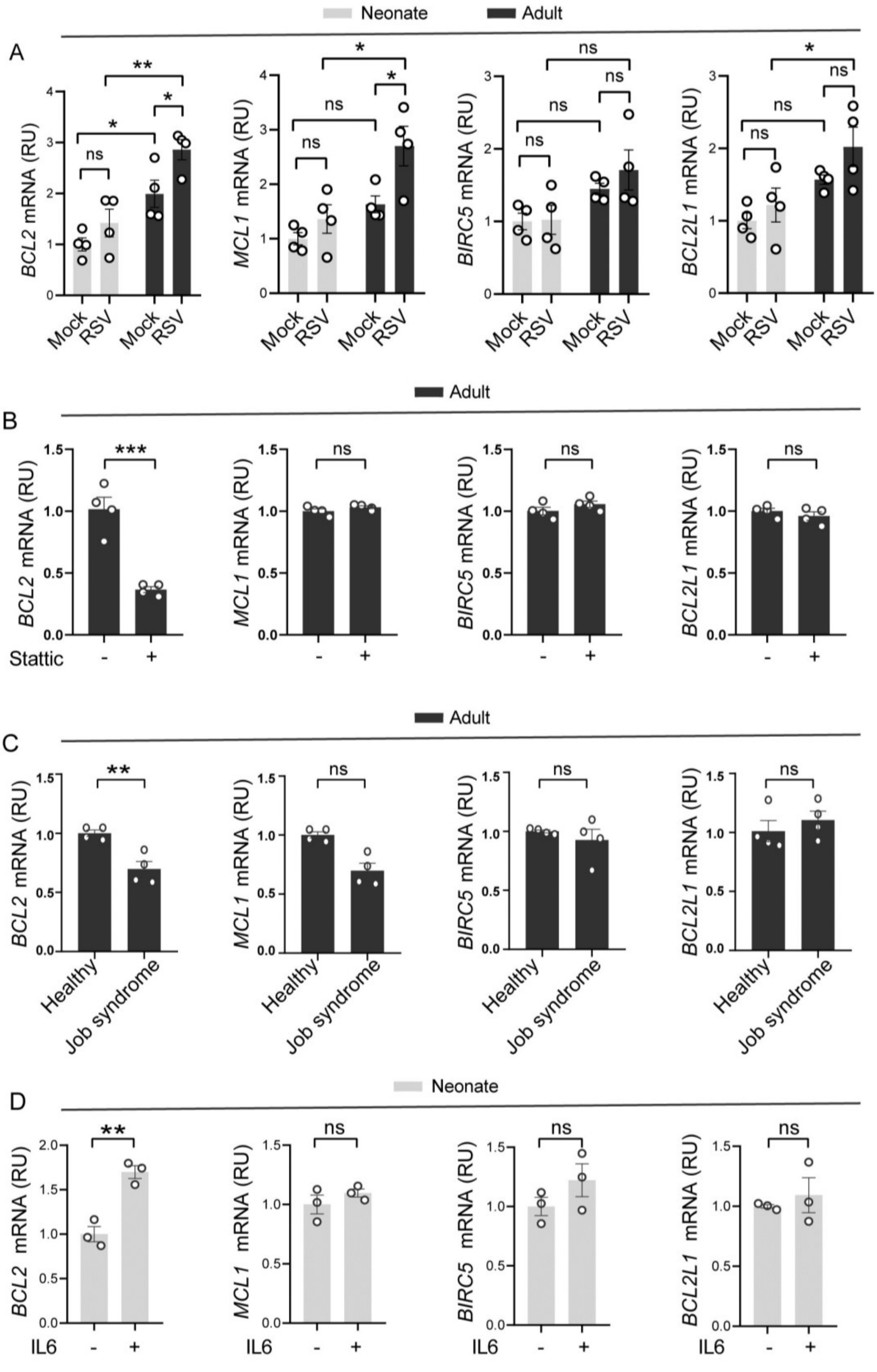

Figure S9. *BCL2* family gene expression associated with RSV infection and STAT3 signaling in neonatal and adult ALI cultures. Related to Figures 7, 8, 9 and S12.

(A-D) Relative levels of *BCL2*, *MCL1*, *BIRC5*, and *BCL2L1* expression in neonatal and adult ALI cultures with and without RSV infection in (A), Stattic (20 μM)-treated adult ALI cultures in (B), adult ALI cultures of BSCs from healthy donors and a patient with Job syndrome harboring STAT3-S560del mutation (C), neonatal ALI cultures with and without IL6 (50 ng/mL) treatment in (D).

Each dot represents one BSC line in (A) or one of 4 independent experiments of one BSC line in (B, C, and D). Bar graphs show mean ± SEM. *p<0.05, **p<0.01, and ***p<0.001 calculated by two-way ANOVA followed by Dunn's test in (A) and by Student's t-test (two-tailed) in (B-D).

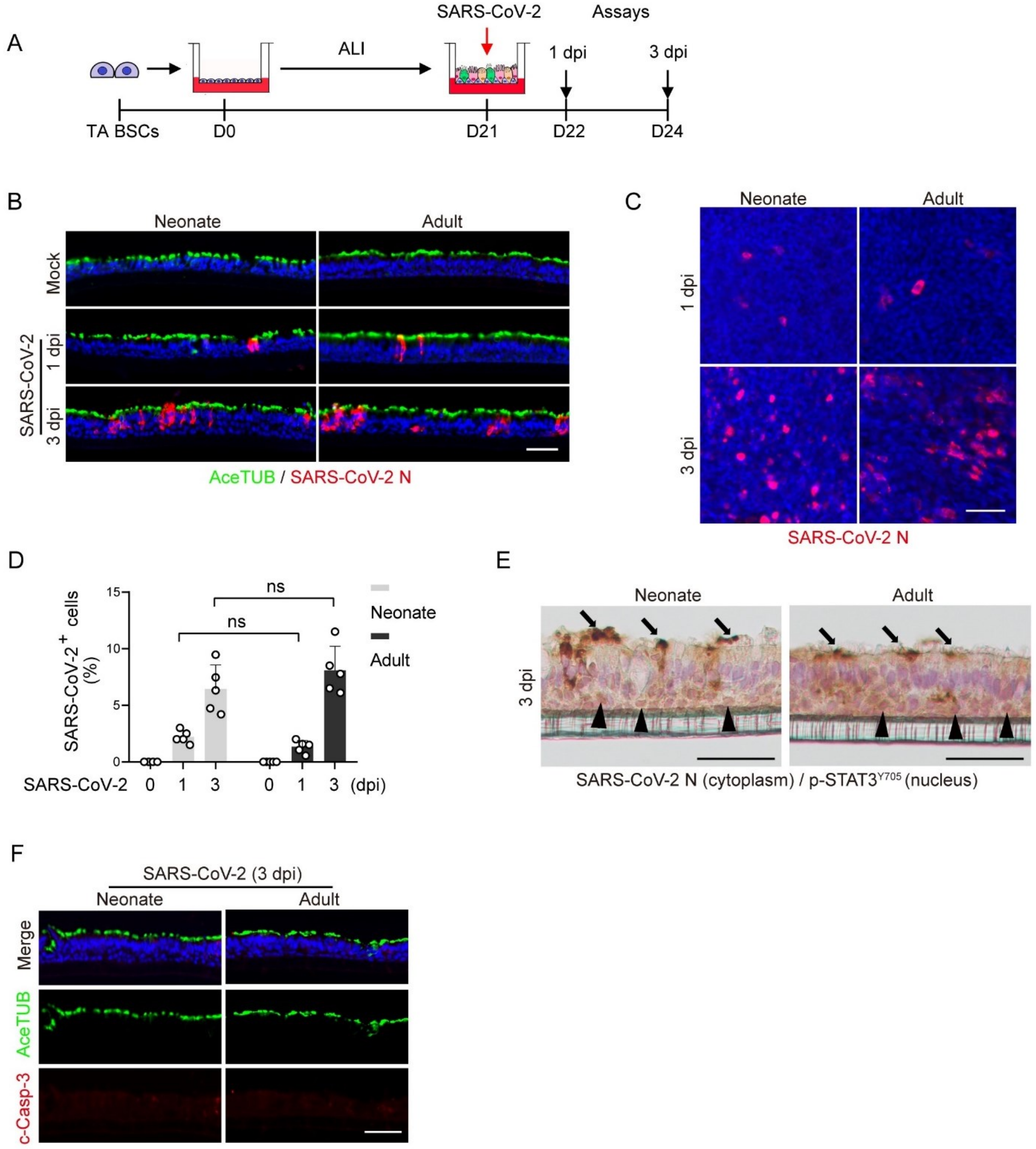

Figure S10. Age has no effect on SARS-CoV-2 infection of human bronchial epithelium. Related to Figures 2 and 7.

(A) Schematic of SARS-CoV-2 infection (MOI 3) of day 21 ALI cultures of neonatal and adult TA BSCs (n=2 donors) followed by analyses at 1 and 3 dpi. (B) Representative double staining for AceTUB and SARS-CoV-2 N in cross sections of ALI cultures. (C) Representative whole-mount staining for SARS-CoV-2 N. (D) The relative abundance of SARS-CoV-2 N^{+} cells in neonatal and adult ALI cultures. Each dot represents quantification of one 20X image of ALI cultures from 2 donors. (E) Representative chromogenic double staining for SARS-CoV-2 N (cytoplasm) and p-STAT3^{Y705} (nucleus) at 3 dpi. Arrows point to SARS-CoV-2 N^{+} ciliated cells. Arrowheads point to cells positive for nuclear p-STAT3^{Y705}. (F) Representative

double staining for c-Casp-3 and AceTUB at 3 dpi.

Each data point (D) represents quantifications of one 20X image of one BSC line. Bar graph represents mean ± SEM. ns, not significant by two-way ANOVA followed by Dunn’s test. Similar results were observed in ALI cultures of 2 donors. Scale bars, 50 μm.

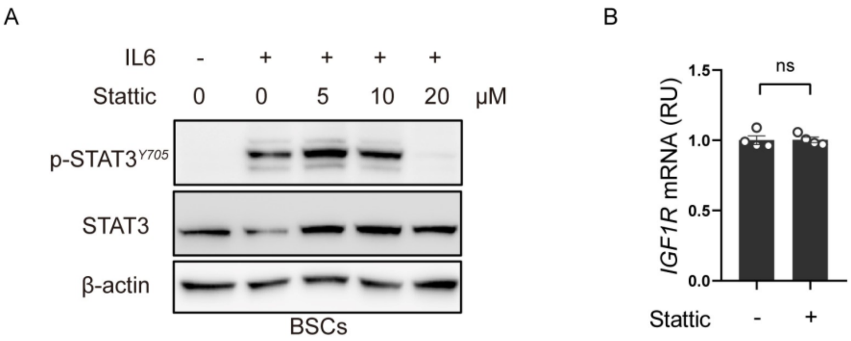

Figure S11. Dose-dependent blockade of IL6-induced STAT3 activation by Stattic. Related to Figure 8.

(A) Representative Western blot assay for the level of p-STAT3^{Y705} and STAT3 in BSCs pretreated with Stattic at different concentrations 2 h prior to IL-6 stimulation (50 ng/mL). Samples were collected 30 min after IL6 stimulation. β-actin was loading control. (B) Relative levels of *IGF1R* gene expression in adult ALI cultures with and without Stattic treatment for 48 hours by RT-qPCR. Stattic (20 μM) was given to the bottom chamber.

Each dot represents one experiment. Bar graphs show mean ± SEM. ns, not significant by Student's t-test (two-tailed).

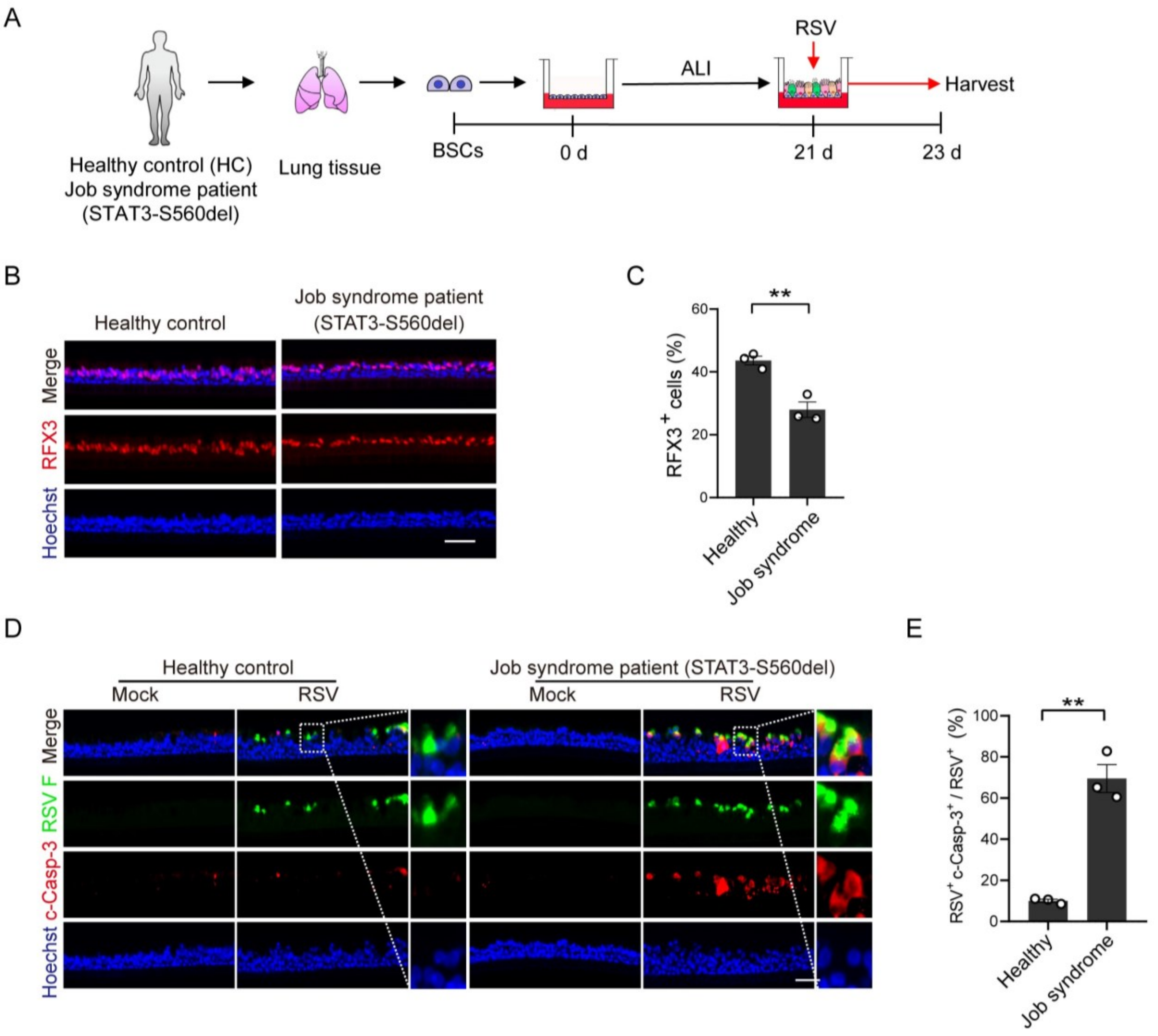

Figure S12. ALIs culture derived from BSCs from an adult patient with Job syndrome shows worsened apoptosis following RSV infection. Related to Figure 8.

(A) Schematic of ALI cultures generated with BSCs isolated from lung biopsy samples of healthy donors and a patient with Job syndrome harboring STAT3-S560del mutation. (B) Representative RFX3 staining in healthy and Job syndrome ALI cultures. (C) The relative abundance of RFX3$^+$ ciliated in healthy and Job syndrome ALI cultures. (D) Representative double staining for RSV F and c-Casp-3 in healthy and Job syndrome ALI cultures at 2 dpi. (E) The relative abundance of RSV F$^+$c-Casp-3$^+$ double positive cells in RSV$^+$ cells in these cultures.

Data points represent 3 independent experiments of one BSC line for each group in (C and E). Bar graphs show mean ± SEM. **p<0.01 calculated by Student's t-test (two-tailed). Scale bars, 50 μm.

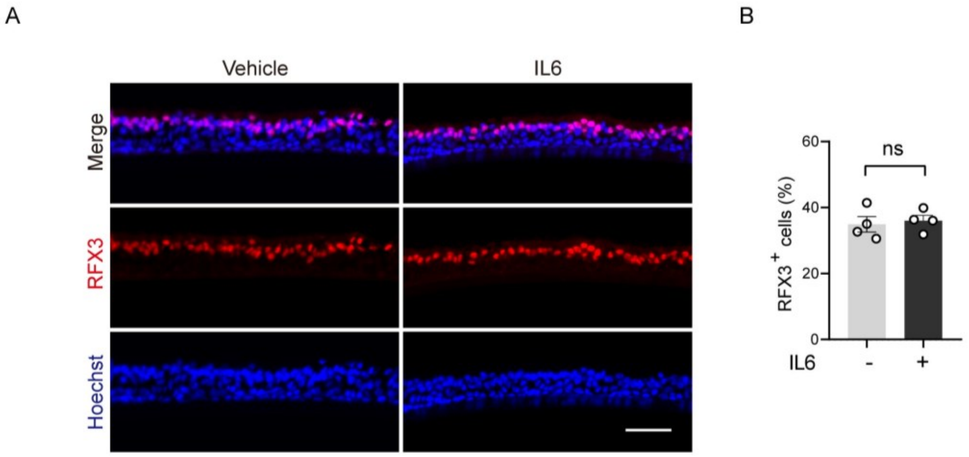

Figure S13. IL6 treatment starting from day 18 in ALI has no effect on ciliated cell differentiation. Related to Figure 9.

(A) Representative RFX3^{+} staining in ALI cultures treated with vehicle or IL6 (50 ng/mL) starting from day 18 in ALI. ALI cultures were assayed at day 21. (B) The relative abundance of RFX3^{+} ciliated with and without IL6 treatment.

Data points represent 4 independent experiments of one neonatal BSC line. Bar graph shows mean ± SEM. ns, not significant calculated by Student's t-test (two-tailed). Scale bar, 50 μm.

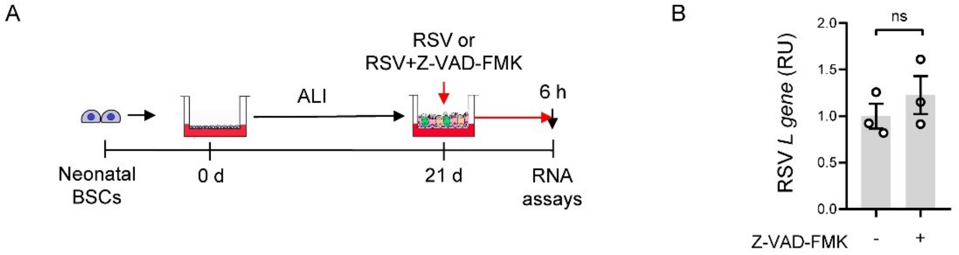

Figure S14. Mixing RSV with Z-VAD-FMK has no effect on RSV infection. Related to Figure 10.

(A) Schematic of infection by mixing Z-VAD-FMK (40 μM) with RSV. RSV mixed with/without Z-VAD-FMK was applied in the apical chamber. The assay was performed at 6 hpi. (B) Relative levels of RSV *L* gene at 6 hpi by RT-qPCR.

Bar graph shows mean ± SEM. ns, not significant calculated by Student's t-test (two-tailed).

REFERENCES

1. Wagner R, Amonkar GM, Wang W, Shui JE, Bankoti K, Tse WH, et al. A Tracheal Aspirate-Derived Airway Basal Cell Model Reveals a Proinflammatory Epithelial Defect in Congenital Diaphragmatic Hernia. *Am J Respir Crit Care Med.* 2023.
2. Huang J, Hume AJ, Abo KM, Werder RB, Villacorta-Martin C, Alysandratos KD, et al. SARS-CoV-2 Infection of Pluripotent Stem Cell-Derived Human Lung Alveolar Type 2 Cells Elicits a Rapid Epithelial-Intrinsic Inflammatory Response. *Cell Stem Cell.* 2020;27(6):962-73 e7.
3. Amonkar GM, Wagner R, Bankoti K, Shui JE, Ai X, and Lerou PH. Primary culture of tracheal aspirate-derived human airway basal stem cells. *STAR Protoc.* 2022;3(2):101390.
