# Supplementary material for: Activation of STAT3-mediated ciliated cell survival protects against severe infection by respiratory syncytial virus
Source: J Clin Invest. 2024 Nov 1;134(21):e183978. doi: 10.1172/JCI183978 (PMC11527452; doi:10.1172/JCI183978)

Full unedited gel for **Figure 2C**

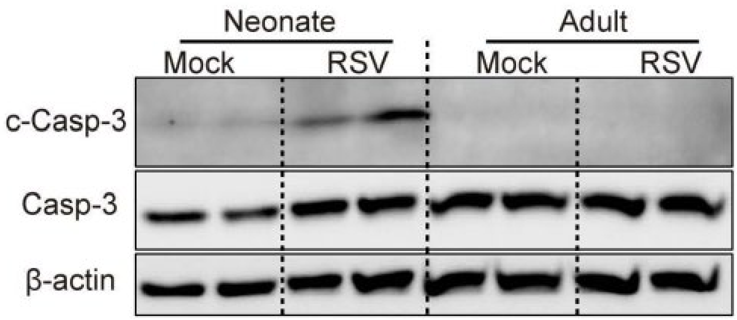

C-Casp-3

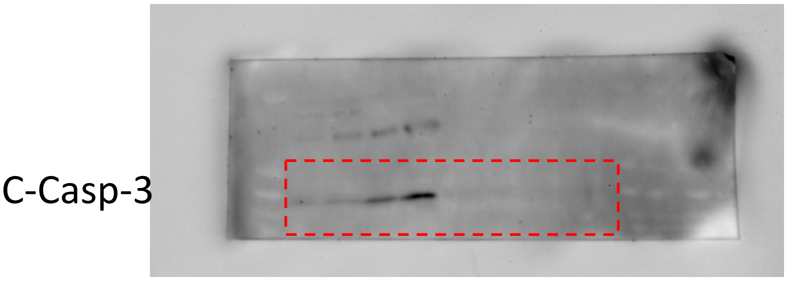

Casp-3

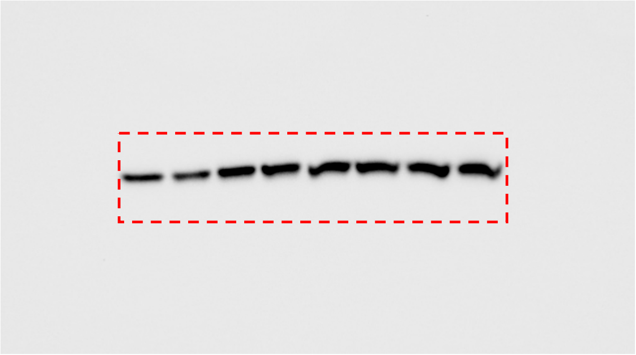

Actin

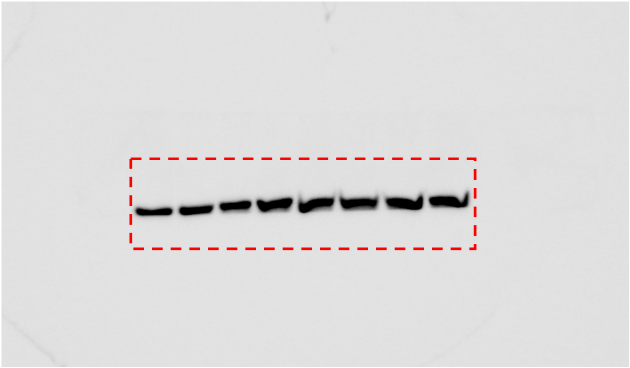

Full unedited gel for **Figure 5A**

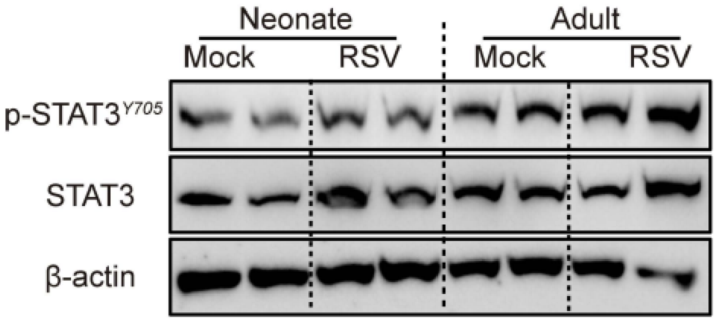

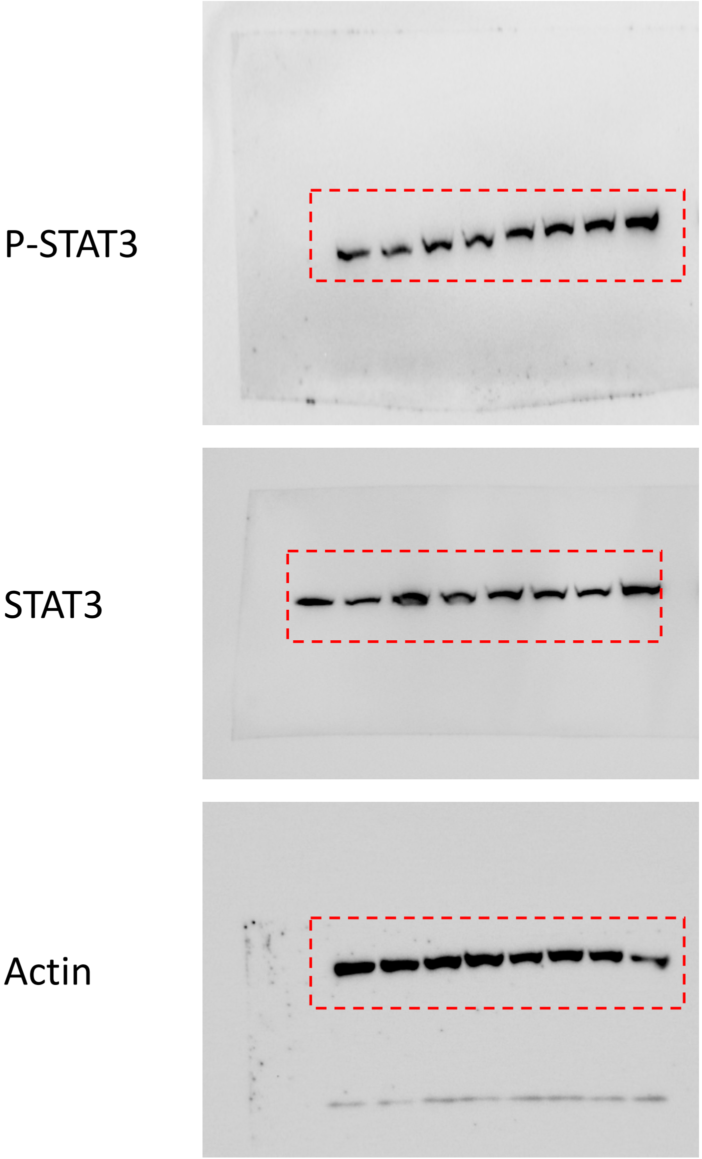

Full unedited gel for **Figure 6F**

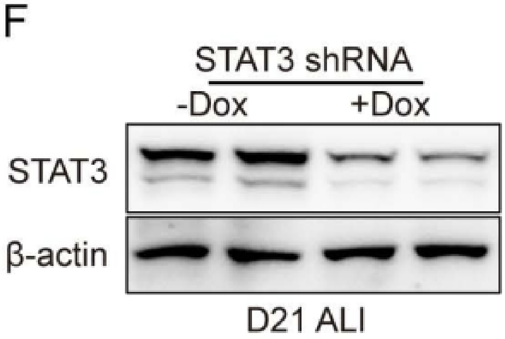

STAT3

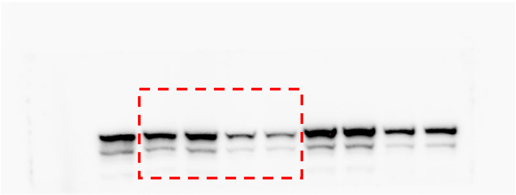

Actin

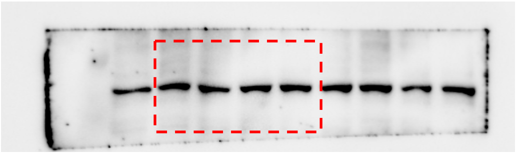

Full unedited gel for **Figure 7B**

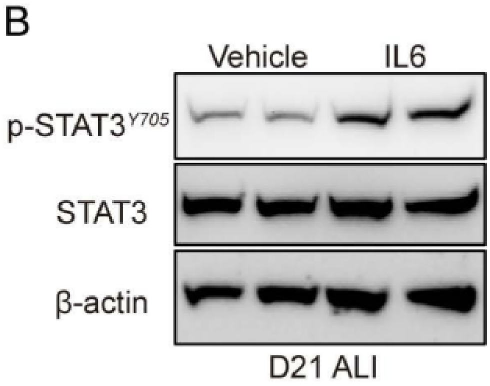

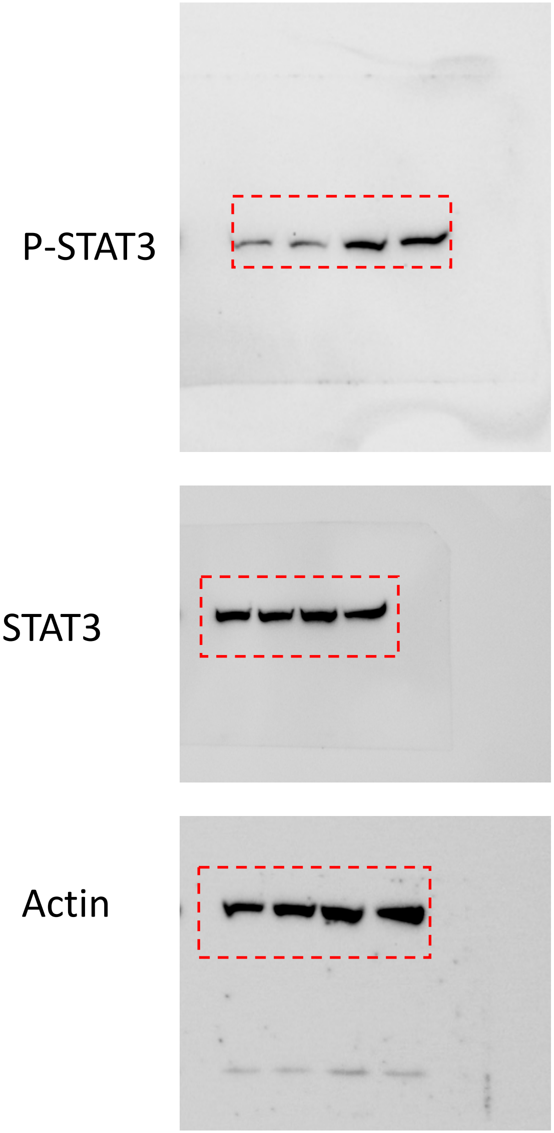

Full unedited gel for **Figure S6G**

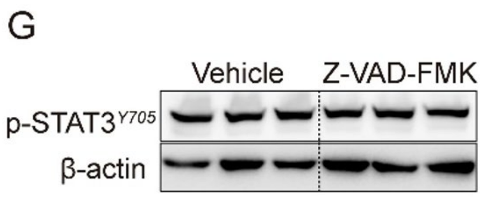

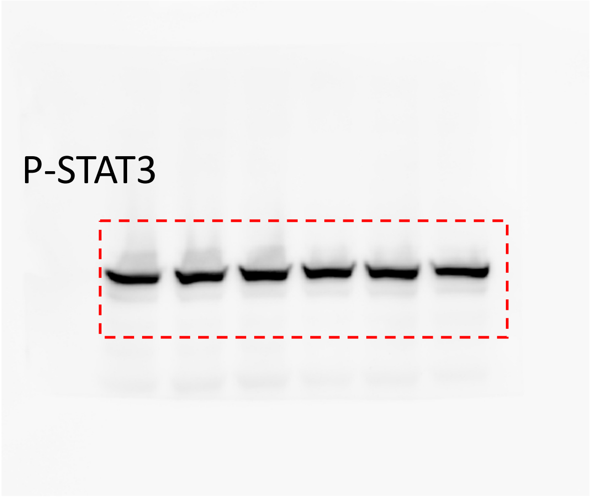

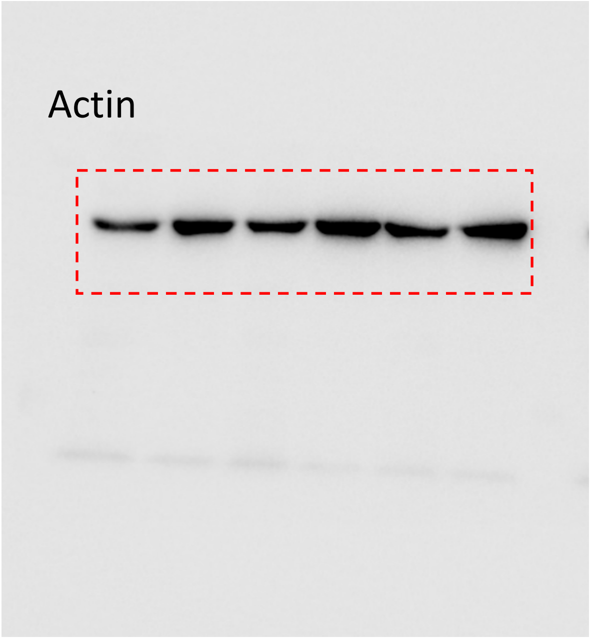

Full unedited gel for **Figure S11A**

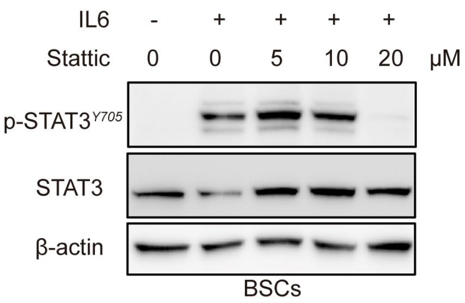

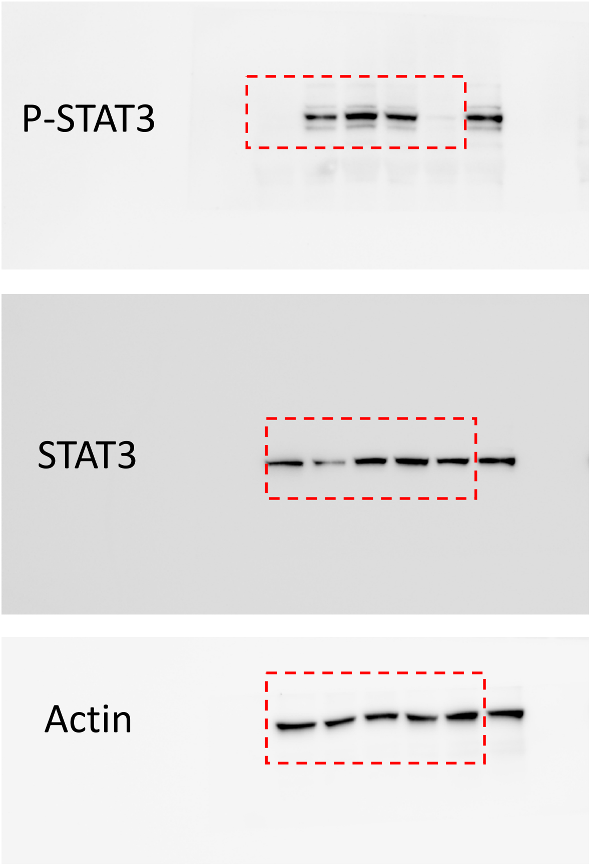

Supplement: Unedited blot and gel images [file jci-134-183978-s013.pdf]
